# Discovery of intrahepatic CD103^+^ cDC1/CD8^+^ T_RM_ protective immune axis against acetaminophen-induced acute liver injury

**DOI:** 10.1038/s12276-025-01565-3

**Published:** 2025-11-07

**Authors:** Jinjoo Lee, Myeong-Ho Kang, Kee-Hyun Kwon, Min-Suk Cha, JungHyub Hong, Yoe-Sik Bae, Seok-Hee Park, Siyoung Yang, Hye Young Kim, Kyung Chul Yoon, Yong-Soo Bae

**Affiliations:** 1 Department of Biological Science, Center for Immune Research on Nonlymphoid Organs, Sungkyunkwan University, Suwon, Republic of Korea; 2 Center for Immune Research on Nonlymphoid Organs, Sungkyunkwan University, Suwon, Republic of Korea; 3 Department of Health Sciences and Technology, Samsung Advanced Institute for Health Sciences and Technology, Sungkyunkwan University, Seoul, Republic of Korea; 4 Laboratory of Mucosal Immunology, Department of Biomedical Sciences, Seoul National University College of Medicine, Institute of Allergy and Clinical Immunology, Seoul National University Medical Research Center, Seoul, Republic of Korea; 5 Institute of Allergy and Clinical Immunology, Seoul National University Medical Research Center, Seoul, Republic of Korea; 6 Department of Surgery, SMG-SNU Boramae Medical Center, Department of Surgery, Seoul National University, College of Medicine, Seoul, Republic of Korea; 7 Department of Surgery, Seoul National University, College of Medicine, Seoul, Republic of Korea

**Keywords:** Translational immunology, Innate immunity

## Abstract

Understanding the intrahepatic protective immune systems against acetaminophen (APAP)-induced acute liver injury (ALI) is currently limited. Here we reveal that Gram-positive gut-microbiota-derived pathogen-associated molecular patterns promote the CCL2-dependent infiltration of hepatotoxic Ly6C^hi^ monocytes into the APAP-damaged liver, thus inducing APAP-ALI. Conversely, Gram-negative bacterial pathogen-associated molecular patterns activate hepatic CD103⁺ cDC1s to produce IL-15, which in turn expands intrahepatic tissue-resident memory CD8⁺ T (T_RM_) cells and promotes protective immunity against APAP-derived liver injury. APAP-ALI was further exacerbated in Batf3-knockout and Rag1-knockout mice owing to an increased population of intrahepatic Ly6C^hi^ monocytes in both knockout strains. The adoptive transfer of hepatic CD8^+^ T cells or hepatic CD103^+^ cDC1s from wild-type mice ameliorated APAP-ALI in both knockout mice. Notably, CD44^+^CD69^+^ T_RM_ cells within hepatic CD8^+^ T cells, when activated by IL-15/IL-15Rα from hepatic CD103^+^ cDC1s of APAP mice, played a crucial role in inducing apoptosis of liver-infiltrating monocytes through direct cell-to-cell interactions and granzyme B secretion. Human results supported these animal findings. Our findings underscore the existence of an intrahepatic protective immune system, the hepatic CD103^+^ cDC1/CD8^+^ T_RM_ axis, which regulates APAP-ALI by controlling pathogenic monocytes.

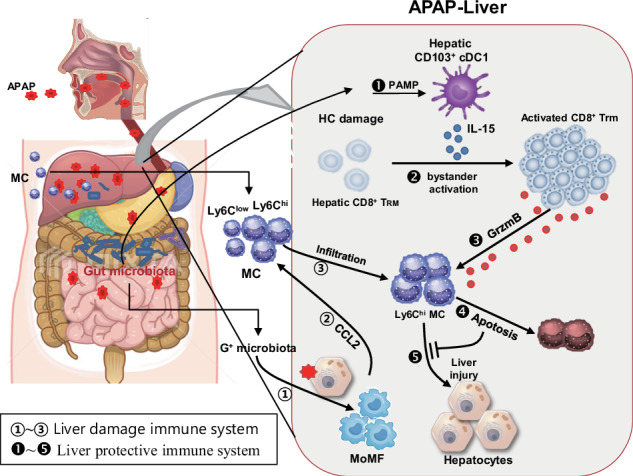

## Gut microbiota shapes liver immunity against acetaminophen injury

Acetaminophen is a common pain reliever, but an overdose can cause severe liver damage, known as acute liver injury. Researchers found that gut bacteria influence how immune cells respond to this damage. Signals from Gram-positive bacteria encouraged harmful monocytes to enter the liver and worsen injury, whereas signals from Gram-negative bacteria activated a protective immune pathway. In this pathway, a special liver immune cell type called cDC1s released the molecule IL-15, which stimulated CD8⁺ tissue-resident memory T cells to destroy the harmful monocytes. This CD103⁺ cDC1/IL-15/CD8⁺ tissue-resident memory T cell ‘protective immune axis’ helped limit liver damage in mice, and similar results were supported by human data. The study highlights a promising new therapeutic direction for treating acetaminophen-induced liver injury by strengthening the liver’s own protective immune system.

This summary was initially drafted using artificial intelligence, then revised and fact-checked by the author.

## Introduction

Acetaminophen (APAP) is the most used antipyretic and analgesic drug. APAP is safe and effective when used at the recommend dose, but overdose causes hepatotoxicity, sometimes leading to acute liver injury (ALI). APAP-ALI can be treated with the antioxidant *N*-acetyl cysteine, but it is only effective within the first 10 h during the initial stage of liver failure^1–3^. If early treatment is missed, damage from ALI may be life-threatening or require a liver transplantation^2^.

In general, the oxidative stress and mitochondrial dysfunction caused by *N*-acetyl-*p*-benzoquinone imine, a toxic byproduct of APAP, play an important role in the pathogenesis of APAP-ALI^1,4^. The detailed immunological mechanism of hepatotoxicity caused by APAP has been reported as follows: the hepatotoxicity of APAP adducts is amplified by proinflammatory immune cells. When APAP is overdosed, hepatocytes undergo necrosis due to oxidative stress and mitochondrial damage, resulting in the secretion of damage-associated molecular patterns (DAMPs), which are recognized by Kupffer cells (KCs) and/or monocyte-derived macrophages (MoMFs)^5,6^. This leads to the activation of KCs and MoMFs secreting proinflammatory cytokines, reactive oxygen species and chemokines, ultimately resulting in the infiltration of neutrophils, monocytes and/or natural killer (NK) cells into the liver^7–9^. These innate immune cells migrated to the liver amplify liver cell necrosis by secreting proinflammatory cytokines^9–11^.

Dendritic cells (DCs) are notable among innate immune cells for their unique properties. Conventional DCs (cDCs) play an important role in priming T cells by serving as a bridge between innate and adaptive immunity. cDCs in nonlymphoid organs are commonly classified into two subsets on the basis of their genetic profile: CD103^+^ cDC1 (which cross-presents antigen to CD8^+^ T cells via MHC-I) and CD11b^+^ cDC2 (which presents MHC-II-bound antigens to CD4^+^ T cells). Organ-resident DCs determine the balance between immune tolerance and immunogenicity. In particular, hepatic CD103^+^ cDC1 has a protective role against liver fibrosis^12–14^, hepatocellular carcinoma (HCC)^15^ and inflammation after liver transplantation^16^. So far, only two reports have been published on the role of DCs in ALI models with APAP^17,18^. Connolly et al. showed that DC depletion in CD11c-DTR mice exacerbated APAP-ALI^17^. However, the detailed immunological mechanisms remain unanswered. Recently, Hildreth et al. demonstrated that in cases of ALI, cDC1s induce the secretion of interferon-γ (IFN-γ) from liver-resident innate lymphoid cell type 1 through IL-12 production, thereby preventing hepatocyte necrosis^18^. However, this claim and their previous results^19^ are accompanied by a controversial aspect due to the well-established understanding that IFN-γ generally exacerbates liver injury instead of alleviating it^20–22^.

T cells are also known to exacerbate APAP-ALI^23^. In addition to APAP, concanavalin A, *Pseudomonas aeruginosa* exotoxin A and carbon tetrachloride are also known to induce liver damage in a T cell-dependent manner when inoculated intraperitoneally (i.p.)^24,25^. These reports suggest that TNFα and IFN-γ secreted from CD4^+^ T cells exacerbate ALI. Contrary to these reports, many studies revealed that hepatic T cells, mainly hepatic tissue-resident memory CD8^+^ T (T_RM_) cells play a protective role instead of inducing liver injury in viral and parasite infection^26,27^, nonalcoholic fatty liver disease^28^, HCC^29^ and liver transplantation^30^.

Research on the interaction between gut microbiota and liver diseases has been active in recent years. Dysbiosis has been shown to exacerbate liver disorders such as nonalcoholic fatty liver disease^31^, alcoholic steatohepatitis^32^, liver fibrosis^33^ and HCC^34,35^. In addition, it has been reported that the dysbiosis of gut microbiota is closely related to APAP-ALI^36^, and gut microbiota mediates the diurnal variation of APAP-ALI, which can be alleviated by antibiotic administration^37^. Despite evidence suggesting that immune cell activation and intestinal microbes increase APAP-ALI, the interaction between gut microbiota and immune cells in the liver remains poorly understood.

We aimed to elucidate the detailed immunological mechanisms underlying liver damage and its regulation in relation to the interaction between the gut microbiota and immune systems during APAP-ALI. Here, we found that APAP triggers liver damage, followed by the infiltration of Ly6C^hi^ monocytes into the liver with the aid of gut microbiota, leading to the exacerbation of liver damage. However, liver-resident CD8^+^ T_RM_ cells, which are activated by IL-15 secreted from hepatic CD103^+^ cDC1s, regulated monocyte-mediated liver damage by inducing the granzyme B (GrzmB)-mediated apoptosis of the infiltrated Ly6C^hi^ monocytes via cell-to-cell interaction.

## Materials and methods

### Mice

Wild-type (WT) C57BL/6 was purchased from DBL. Batf3 KO (B6.129S(C)-Batf3tm1Kmm/J), Rag1 KO (B6.129S7-Rag1tm1Mom/J) and OT-I (C57BL/6-Tg (TcraTcrb)1100Mjb/J) mice were purchased from Jackson Laboratory. Mice of 6–10 weeks of age were used in the present studies. All mice were inbred and maintained in a specific pathogen-free facility at Sungkyunkwan University following the institute/university animal care and use guidelines. Male mice were used for all analyses, except for those examining sex differences.

### Patients and study samples

This study included ten patients with HCC and other liver diseases who underwent liver resection at Boramae Hospital (Seoul, South Korea). The information of the ten patients is summarized in Table 1. The study adhered to the principles of the Helsinki Declaration and received approval from the institutional review board of Seoul National University Boramae Medical Center (SMG-SNU IRB 20-2023-73). Before enrolling in the study, written consent was obtained from each patient. From the resected liver tissue, adjacent noncancerous healthy tissue was collected, minced and enzymatically digested with collagenase VI (Sigma-Aldrich) to obtain a single cell suspension. Lymphocytes were collected by percoll (Sigma-Aldrich) density gradient centrifugation. Flow cytometry analysis and quantitative real-time PCR were performed on the liver immune cells.

**Table 1:** Patient’s clinical information.

Patients	Sex	Age (years)	Indication for liver resection	Note
Pt 1	M	57	Decompensated LC	Alcoholic
Pt 2	M	79	HCC	Nonviral
Pt 3	F	75	HCC	HCV
Pt 4	M	88	HCC	Nonviral
Pt 5	M	76	HCC	Alcoholic
Pt 6	M	52	HCC	HBV
Pt 7	F	54	HCC	HBV
Pt 8	F	60	Colorectal liver metastasis	Nonviral
Pt 9	M	62	HCC	Nonviral, alcoholic
Pt 10	M	79	Intrahepatic cholangiocarcinoma	Nonviral

Pt, Patient; M, male; F, female; LC, liver cirrhosis; HBV, hepatitis B virus; HCV, hepatitis C virus.

### APAP administration

After 15 h of fasting, 400 mg/kg of APAP (Sigma-Aldrich) was administered orally or injected i.p., and serum was collected through retro-orbital blood sampling at 3, 6, 12 and 24 h after APAP administration. Following APAP administration, mice were killed at each time point, and the immune cells in the liver were analyzed as previously described^38^.

### Administration of ABXs or drug

A mixture of antibiotics (ABXs) including ampicillin (1 g/l), neomycin (1 g/l), metronidazole (1 g/l) and vancomycin (0.5 g/l) and individual antibiotic such as cefoperazone (0.5 g/l) were given to mice through drinking water for 2 weeks. Alternatively, a higher concentration of ABXs (hABXs, 15 mg/kg) was orally administered to the mice either concurrently or with a time difference from the administration of APAP. Peptidoglycan (PGN) was injected i.p. into antibiotic-treated mice at 10 mg/kg in a volume of 100 μl. FTY720 (2-amino-2-[2-(4-octylphenyl)ethyl]propane-1,3-diol) was administered through drinking water at 2 μg/ml for 7 days as previously described^39^.

### Blocking antibodies

For CD8^+^ T cell depletion, mice were i.p. injected with anti-CD8α antibody (200 μg per mouse, clone YTS 169.4). For IL-12, IL-15, IL-18 and CCL2 neutralizing, mice were i.p. injected with 200 μg per mouse of anti-IL-12 antibody (clone R1-5D9), anti-CD122 (IL-15Rβ) antibody (clone TM-Beta1), anti-IL-18 antibody (clone YIGIF74-1G7) and anti-CCL2 antibody (clone 2H5) (all from BioXCell).

### Histology

The liver tissue was fixed with 10% buffered formalin and then embedded in paraffin, and 3-μm sections were cut and stained with hematoxylin and eosin (H&E). The H&E staining slides were used for necrosis and the percent of necrotic area was evaluated through Image J program.

### Serum ALT and AST

The levels of serum alanine aminotransferase (ALT) and aspartate aminotransferase (AST) were measured by the kinetic assay method using the Asan set GOT Assay kit and Asan set GPT Assay kit (Asanpharm) according to the manufacturer’s protocol.

### Flow cytometry

The cells were analyzed by FACSCantoII (BD Bioscience) and sorted using FACSAria Fusion (BD Bioscience) as described previously^40,41^. Data analysis was performed using FlowJo software version 10. All cells were suspended with fluorescence-activated cell sorting (FACS) flow buffer (BD Bioscience) and stained in the dark. The cells were stained for viability with Fixable Viability Dye eFluor506 (Invitrogen) for 15 min at 4 °C. For hepatic DC analysis, the following antibodies were used: I-A/I-E (clone M5/114.15.2), CD103 (clone 2E7), CD3 (clone 17A2), CD19 (clone 1D3), NK1.1 (clone PK136), CD11c (clone N418), CD45.2 (clone 104) and CD11b (clone M1/70) from BioLegend. The T cell analysis was performed with the following antibodies: CD3 (clone 17A2), CD69 (clone H1.2F3), CD4 (clone GK1.5), CD8α (clone 53-6.7), CD62L (clone MEL-14) and CD45.1 (clone A20) from BioLegend and CD44 (clone IM7) and KLRG1 (clone 2F1) from eBioscience. Antibodies against NKG2D (clone C7), NKp46 (clone 29A1.4), CXCR6 (clone SA051D1) and CD49a (clone HMα1) from BioLegend were used for the surface staining of hepatic T_RM_ cells. The intracellular staining for GrzmB (clone GB11) from BioLegend was conducted in the T cells with the BD Cytofix/Cytoperm (BD Biosciences) according to the manufacturer’s protocol, after 4 h of stimulation with 1× activation cocktail (BioLegend). The Foxp3/Transcription Factor Staining Buffer Set (eBioscience) was used to stain Ki67, a proliferation marker, in CD8^+^ T cell subsets. Antibodies against CD11b (clone M1/70), Ly6G (clone 1A8), Ly6C (HK1.4), CD14 (clone Sa14-2) and F4/80 (clone BM8) from BioLegend were used for the detection of neutrophils, monocytes, macrophages and KCs. The intracellular staining for TNFα (clone MP6-XT22), IL-6 (clone MP5-20F3) and CCL2 (clone 2H5) from BioLegend was conducted in the innate immune cells with the BD Cytofix/Cytoperm (BD Biosciences) according to the manufacturer’s protocol, after 4 h of stimulation with 1 μg/ml of lipopolysaccharide (LPS; Sigma-Aldrich) and 1× Brefeldin A (BioLegend). The apoptosis analysis was conducted in the innate immune cells with the FITC Annexin V Apoptosis Detection Kit I (BD Biosciences) according to the manufacturer’s protocol.

### Cell sorting

cDC1s or cDC2s from mouse liver immune cells were identified as CD45.2^+^ lineage (CD3^−^CD19^−^NK1.1^−^), MHC-II^+^CD11c^high^ and CD11b^+^CD103^−^ for cDC2 or CD11b^−^CD103^+^ for cDC1. The T cell subsets from mouse immune liver cells were identified as B220^−^CD3^+^ and CD4^+^CD8^−^NK1.1^−^ for CD4^+^ T cell or CD4^−^CD8^+^NK1.1^−^ for CD8^+^ T cell or CD4^−^CD8^−^NK1.1^+^ for NK T (NKT) cells. The subsets of CD8^+^ T cells were identified as CD62L^−^CD44^+^KLRG1^−^CD69^+^, CD62L^−^CD44^+^KLRG1^+^CD69^−^, CD62L^−^CD44^+^KLRG1^−^CD69^−^ and CD62L^−^CD44^−^. Human hepatic cDC1, cDC2, CD8^+^ T cells and monocytes were identified following the strategy shown in Supplementary Fig. 14. The immune cells identified according to their cell surface markers were sorted through FACS Aria Fusion (BD Bioscience).

### Adoptive transfer

The immune cells were isolated from the liver or the spleen of APAP-treated or untreated donor mice as previously described^38^. The total immune cells or sorted immune cells from donor mice were diluted in phosphate-buffered saline, and 1 × 10^6^ cells (total liver immune cells), 5 × 10^5^ cells (NKT cells, CD4^+^ and CD8^+^ T cells) or 2 × 10^5^ cells (hepatic cDC1s and cDC2s) were adoptively transferred at a total volume of 100 μl to WT or knockout (KO) recipient mice intravenously via the tail vein route.

### Enzyme-linked immunosorbent assay

Enzyme-linked immunosorbent assay kits for measuring CCL2 were purchased from Biolegend (432704). All measurements were performed according to the manufacturer’s protocol.

### Monocyte migration assay

The Ly6C^hi^ monocytes sorted from the peripheral blood mononuclear cell (PBMC) and liver were placed in the upper chamber of the Transwell. In the lower chamber, liver extract diluted one tenth with Roswell Park Memorial Institute 1640 medium (Gibco) and filtered through a 40-μM strainer was placed with or without anti-CCL2 neutralizing antibody. After incubation at 37 °C for 24 h, the number of monocytes that migrated to the lower chamber were counted.

### Cell cytotoxicity assay

The hepatic CD8^+^ T cells and NK cells were isolated from the liver of APAP mice 24 h after the APAP administration. The hepatic CD8^+^ T cells were divided into CD69^−^CD8^+^ T cells and CD69^+^CD8^+^ T cells. The hepatic Ly6C^hi^ monocytes or hepatocytes isolated from the liver of normal WT mice were cocultured with hepatic CD8^+^ T cells or NK cells. The effector and target cells were cocultured for 15 h at a 1:1 ratio (1 × 10^5^ cells) in 96-well plates, and then, cell death was measured using the FITC Annexin V Apoptosis Detection Kit I (BD Biosciences). The coculture experiments were done in the absence or presence of neutralizing antibodies: 10 µg/ml of anti-IFNγ (clone XMG1.2), anti-FasL (clone MFL3), anti-TRAIL (clone MD5-1), anti-CD44 (clone IM7), anti-ICAM-1 (clone YN1/1.7.4), anti-NKG2D (clone CX5 and HMG2D) and anti-NKG2A/C/E (clone 20D5) from Bio X Cell and 0.5 µg/ml of anti-GrzmB (AF1865) from R&D System. Human CD69^+^CD8^+^ T cells and CD14^+^CD16^int^ monocytes were isolated from noncancerous healthy liver tissues obtained from patients with HCC and other liver diseases and then cocultured at 1:0, 1:1, 1:5 and 1:10 ratios in 96-well plates. After 15 h, cell death was measured by annexin V staining.

### Bystander T cell activation analysis using OT-I T cells

For the preactivation of the OT-I T cells, OVA-loaded DCs were injected into OT-I mice, and hepatic OT-I T cells were sorted 60 days later. CD45.2^+^ OT-I T cells were adoptively transferred into CD45.1^+^ WT mice and administered APAP the next day. After 24 h, the proportion of CD45.2^+^ OT-I T cells among the total CD8^+^ T cells and the proportion of T_RM_ among OT-I T cells were examined.

### In vitro culture of hepatic CD8^+^ T cells

The hepatic CD8^+^ T cells were cultured in the presence of 5 ng/ml of IL-2 and with or without 50 ng/ml of IL-15 for 24 h. The hepatic cDC1s were prestimulated with 1 μg/ml of LPS for 3 h. The cells were cocultured in 96-well round-bottom plates at a ratio of 1:5 with or without 10 μg/ml of anti-CD122 (IL-15Rβ) blocking antibody for 24 h, and the frequency of CD69^+^ cells in CD8^+^ T cells was examined.

### IL-15Rα expression on the surface of hepatic cDC1s

The primary mouse hepatocytes or Hepa1-6 cells were treated with 30 μM APAP to induce cell damage, and the supernatants were collected 6 h later. The culture supernatants were used as a source of DAMPs after one fifth dilution to stimulate hepatic cDC1s. In other cases, the hepatic cDC1s were directly treated with 1 μg/ml of LPS, 500 ng/ml of HMGB1 or 6 μM APAP. After 24 h, the cDC1s were assessed for the expression of IL-15Rα by FACS.

### Quantitative real-time PCR

Total RNA was extracted from isolated human CD11b^−^ DC1s using RNAiso Plus (TaKaRa, 9109). The reverse transcription was performed by Maxime RT PreMix (iNtRON Biotechnology, 25081). Real-time quantitative PCR was performed by using qGreen Q-PCR Master Mix (GenDEPOT, Q5600).

The primer sequences for PCR amplification were:

*Il15*: GCAAGCTTGTCCAATCACGG (F) TTTGATTTCCCTGGGCCACA (R),

*Il15RA*: CCACCCTCCACAGTAACGAC (F) GCTGTGTTGTTTGAGCTGGG (R) and

*Atcb*: GGCCAGGTCATCACCATTG (F) GGATGTCCACGTCACACTTCA (R).

### Data analysis using published human liver tissue scRNA-seq data

Single-cell RNA sequencing (scRNA-seq) data of liver cells from healthy donor and APAP-induced liver failure patients were collected from database GSE223581. The data analysis was performed using Cellenics (Biomage), a web-based and open-source platform of scRNA-seq analysis. The folder including Feature-Barcode Matrices from each donor was uploaded in Cellenics. Then, the data processing and integration were automatically performed according to Cellenics’ process. The expression of IL-15 level was assessed as average of whole normalized values from each donor. For the T_RM_ ratio, the ratio of CD69 >0.1 was assessed in the CD8A >0.1 population for CD8^+^ T cells. The linear correlation between IL-15 level and T_RM_ ratio was analyzed in the healthy donor samples.

### Statistics

The statistical analysis was performed using Prism software version 10.2.3 (GraphPad). For group statistics, comparison value, one-way and two-way analysis of variance (ANOVA) or the unpaired, two-tailed Student’s *t*-test was used, with significance indicated as *P < 0.05, **P < 0.01, ***P < 0.001 and ‘ns’ denoting nonsignificant differences, in all figures. The quantitative data in this study are expressed as the mean ± s.e.m. The black asterisks represent differences compared with WT mice; red asterisks, differences with Rag1-KO mice; and blue asterisks, differences with Batf3-KO mice.

## Results

### Gut microbiota and activated immune cells exacerbate APAP-induced liver injury

**Fig. 1:**
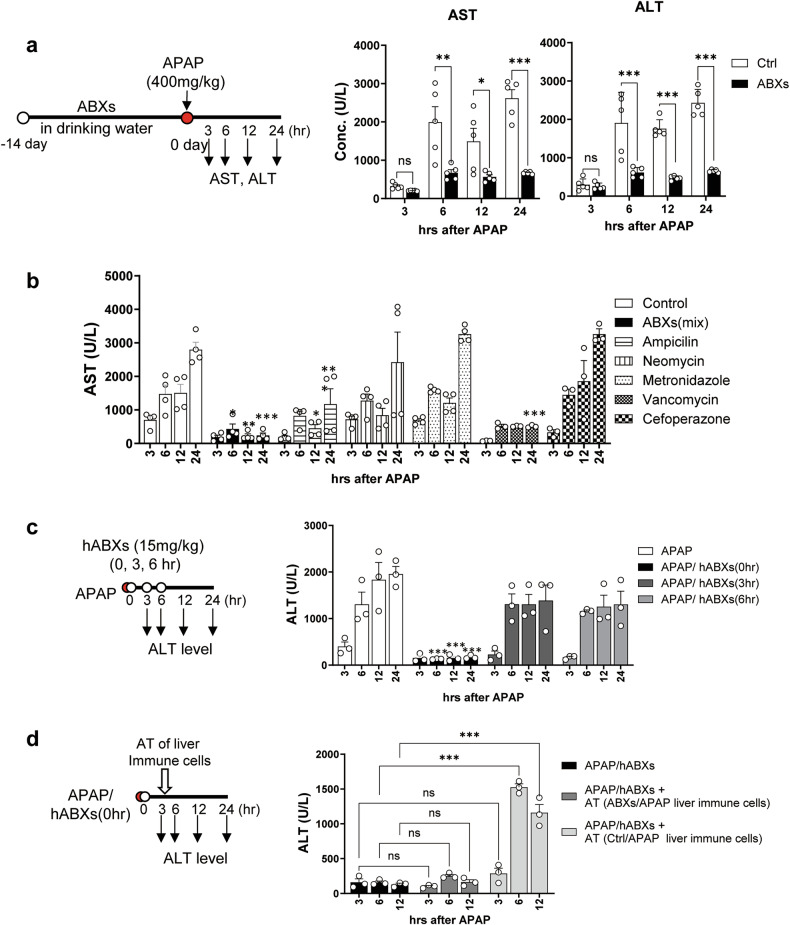
Gut microbiota and hepatic immune cells are involved in the APAP-ALI. Each schematic diagram shows the time of ABXs (white dot) and APAP (red dot) administration and the measuring time of serum AST and ALT level (arrow). **a** The AST and ALT level in mice pretreated with ABXs. *n* = 5 per group per time point. **b** The ALT levels in the mice pretreated with ABXs or individual ABX. *n* = 4–5 per group per time point. **c** The ALT levels of the mice administered with hABXs (15 mg/kg) after the administration of APAP. *n* = 3 per group per time point. **d** The liver immune cells from ABXs-pretreated (ABXs/APAP) or ABXs-untreated APAP (ctrl/APAP) mice were adoptively transferred (AT) to the APAP/hABXs-(0 h)-mice. *n* = 3 per group per time point. Unpaired two-way ANOVA with Šidák correction in **a** and Dunnett in **b**–**d** for posttest were used to measure significance. The error bars indicate mean ± s.e.m. n.s, not significant. *P < 0.05, **P < 0.01, ***P < 0.001.

To examine the impact of gut microbiota on APAP-ALI, mice were pretreated with ABXs in their drinking water for a duration of 2 weeks before oral administration of APAP. In mice preadministrated with ABXs, the levels of serum ALT and AST showed no increase (Fig. 1a). In the further investigation of each antibiotic within the ABXs, it was found that vancomycin exhibited the highest efficacy in inhibiting APAP-ALI, whereas neomycin, metronidazole or cefoperazone had little impact on APAP-ALI in mice (Fig. 1b). These results indicate a strong association between APAP-ALI and gut microbiota, specifically Gram-positive (G^+^) bacteria rather than Gram-negative (G^−^) bacteria, protozoa or normal flora residing in other organs or the skin. Even without the preadministration of ABXs, when mice were simultaneously administered with APAP and hABXs (APAP/hABXs (0 h)), APAP-ALI did not occur in APAP mice (Fig. 1c). However, when hABXs were administered 3 h after APAP overdose, liver injury was not controlled (Fig. 1c). By contrast, when liver immune cells isolated from APAP mice were adoptively transferred to hABXs-treated mice, followed by the administration of APAP, the ALT level significantly increased but did not by transfer of the liver immune cells from APAP/hABXs-(0 h)-treated mice (Fig. 1d). These findings indicate that the gut microbiota alone does not directly cause liver damage. Instead, the gut microbiota, particularly intestinal G^+^ bacteria, trigger the activation of immune cells residing in the liver, ultimately leading to the development of APAP-ALI.

### Hepatic CD8^+^ T cells regulate APAP-induced liver injury

**Fig. 2:**
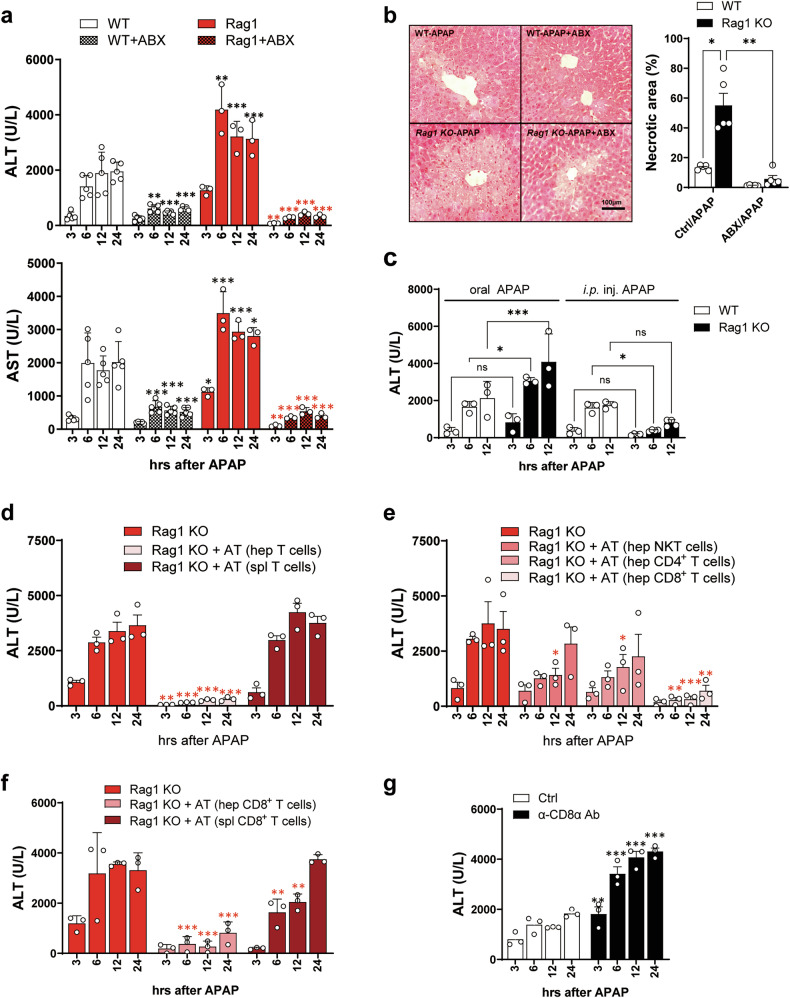
Hepatic CD8^+^ T cells play a protective role in the liver of APAP mice. **a** The serum ALT and AST levels of WT and Rag1-KO APAP mice, **b** The H&E-stained liver sections and necrotic area of WT and Rag1-KO APAP mice pretreated with ABXs. *n* = 3–5 per group per time point. **c** The ALT levels of mice administered orally or i.p. injection (inj.) with APAP. *n* = 3–4 per group per time point. **d**–**f** The ALT levels in Rag1-KO recipient mice adoptively transferred (AT) before APAP administration with hepatic or splenic T cells (**d**); with hepatic NKT cells, CD4^+^ T cells or CD8^+^ T cells (**e**); or with hepatic or splenic CD8^+^ T cells (**f**) from WT normal mice. *n* = 3 per group per time point, respectively. **g** The ALT level of WT mice injected with anti-CD8α antibody before APAP administration. *n* = 3 per group per time point. Unpaired two-way ANOVA with Tukey in **a**–**c**, Dunnett in **d**–**f** and Bonferroni in **g** for the posttest was used to measure significance. The error bars indicate mean ± s.e.m. The black and red asterisks represent differences by time point compared with WT and Rag1-KO mice, respectively. n.s, not significant. *P < 0.05, **P < 0.01, ***P < 0.001.

Taking into account a previous report demonstrating that APAP-ALI worsened due to IFN-γ^+^ CD4^+^ T cells^23^, we initially anticipated that the APAP-ALI would be alleviated in Rag1-KO mice. Unexpectedly, we observed a significant exacerbation of APAP-ALI in Rag1-KO mice compared with WT mice. However, similar to WT mice pretreated with ABXs, the liver injury in Rag1-KO mice was also mitigated by the pretreatment with ABXs (Fig. 2a). Furthermore, the APAP-induced liver necrotic area of Rag1-KO mice was considerably larger compared with that in WT mice, but the necrotic area in ABXs-pretreated Rag1-KO mice was also clearly reduced (Fig. 2b). These results suggest that T cells would be involved in the control of gut-microbiota-mediated APAP-ALI rather than exacerbation. On the basis of a report showing that the toxicity of APAP depends on the route of administration^42^, we examined the APAP-ALI in different administration routes. As reported previously^23^, the intraperitoneal injection of APAP reduced liver damage in Rag1-KO mice compared with WT mice, but oral administration increased liver damage (Fig. 2c). These route-dependent differences may be attributed to distinct immune mechanisms triggered by oral administration versus intraperitoneal injection of APAP. Intraperitoneal injection limits the involvement of gut microbiota and allows direct hepatic exposure, thereby reducing immune-mediated injury in Rag1-KO mice, which lack proinflammatory T cells. By contrast, oral administration engages the gut–liver immune axis, where microbiota-driven signals contribute to T cell-mediated hepatoprotection in WT mice^23^, a response absent in Rag1-KO mice. This may explain the exacerbated liver damage observed in Rag1-KO mice following oral APAP administration. Further studies are warranted to delineate the mechanisms underlying route-dependent immune responses in APAP-induced liver injury. As APAP-induced acute liver failure in many developed countries is an event caused by ingestion of the drug, we adopted the oral administration route in mice. Next, we adoptively transferred T cells into Rag1-KO mice before the oral administration of APAP to determine whether T cells are involved in the control of APAP-ALI or not. APAP-ALI was fully suppressed when the hepatic T cells were adoptively transferred into Rag1-KO mice before APAP administration, whereas they were not suppressed by the transfer of splenic T cells (Fig. 2d). Among the hepatic T cells of APAP mice, only CD8^+^ T cells controlled the APAP-ALI in recipient Rag1-KO mice, whereas hepatic CD4^+^ T cells and NKT cells had a minor effect (Fig. 2e). Furthermore, the adoptive transfer of splenic CD8^+^ T cells was not effective in controlling APAP-ALI in recipient Rag1-KO mice (Fig. 2f). Consistently, the depletion of CD8^+^ T cells with α-CD8α antibodies increased ALT levels in WT APAP mice (Fig. 2g). These results demonstrate that liver-resident CD8^+^ T cells, among the hepatic immune cells in orally administered APAP mice, are involved in protecting the liver from APAP-ALI.

### Hepatic CD103^+^ cDC1s control APAP-ALI by activating hepatic CD8^+^ T cells

**Fig. 3:**
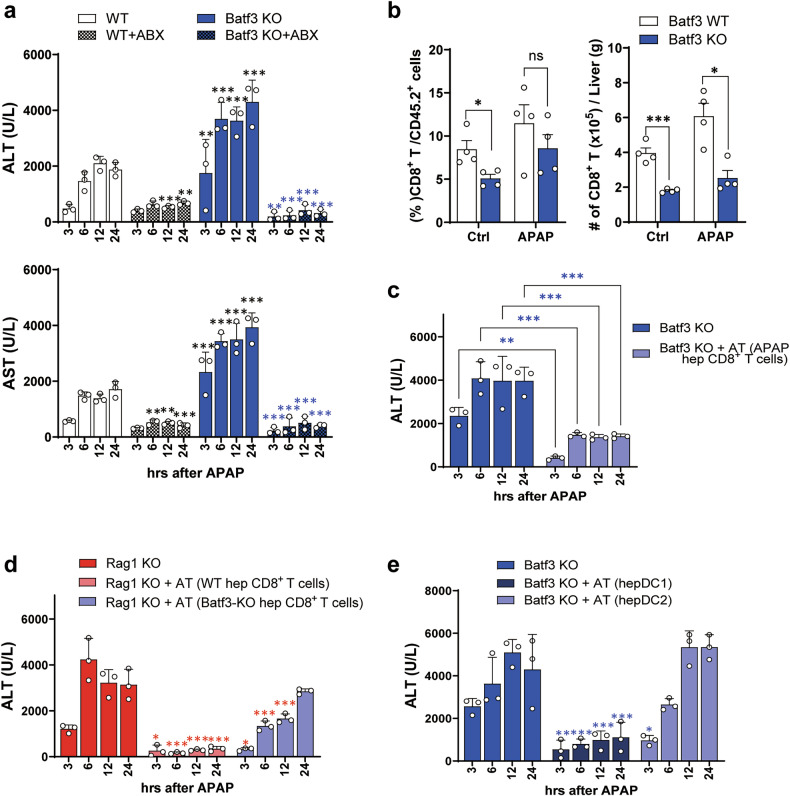
Hepatic CD103^+^ cDC1/CD8^+^ T cell axis plays a crucial role in protecting liver from APAP-ALI. **a** The ALT and AST levels in WT and Batf3-KO mice pretreated with ABXs before APAP administration. *n* = 3 per group per time point. **b** The frequency and number of CD8^+^ T cells in the liver of WT and KO mice. *n* = 4 per group. **c** The ALT levels in Batf3-KO recipient mice adoptively transferred (AT) with WT APAP hepatic CD8^+^ T cells before APAP. *n* = 3 per group per time point. **d** The ALT level of Rag1-KO recipient mice AT with WT or Batf3-KO hepatic CD8^+^ T cells before APAP. *n* = 3 per group per time point. **e** The ALT levels in Batf3-KO recipient mice AT with hepatic DC1s or DC2s of WT mice before APAP administration. *n* = 3 per group per time point. Unpaired two-way ANOVA with Tukey in **a**, Bonferroni in (**c**) and Dunnett in **d**–**e** for the posttest and multiple unpaired *t*-test with Welch’s correction in (**b**) were used to measure significance. The error bars indicate mean ± s.e.m. The black, blue and red asterisks represent the differences by time point compared with WT, Batf3-KO and Rag1-KO mice, respectively. ns, not significant. *P < 0.05, **P < 0.01, ***P < 0.001.

A previous study showed that liver DCs protect against the toxicity of APAP overdose, and their depletion is associated with exacerbated hepatotoxicity^17^. However, a detailed immunological mechanism and DC subset analysis was not clearly demonstrated. As cDC1 is well known to be involved in CD8^+^ T cell priming and activation, we investigated cDC1-deficient Batf3-KO mice. Batf3-KO mice showed severe APAP-ALI compared with WT mice, although the liver injury was also significantly reduced even in Batf3-KO mice when pretreated with ABXs (Fig. 3a). It is widely known that female mice are more resistant than male mice to APAP-ALI^43,44^. However, the severity of APAP-ALI was still more pronounced in Batf3-KO mice than in WT mice, regardless of gender (Supplementary Fig. 1). These results suggest that hepatic CD103^+^ cDC1s also play an important role in suppressing gut microbiota-mediated APAP-ALI, irrespective of gender. The frequency and the number of hepatic CD8^+^ T cells were significantly reduced in Batf3-KO mice (Fig. 3b). The adoptive transfer of WT hepatic CD8^+^ T cells significantly reduced ALT levels in Batf3-KO recipient APAP mice (Fig. 3c). In addition, the adoptive transfer of WT hepatic CD8^+^ T cells to Rag1-KO mice effectively controlled the APAP-ALI of Rag1-KO mice, whereas the adoptive transfer of Batf3-KO hepatic CD8^+^ T cells was little effective (Fig. 3d). These results suggest that APAP-ALI exacerbation in Batf3-KO mice would be due to the absence of hepatic CD103^+^ cDC1-derived CD8^+^ T cells. Consistently, APAP-induced hepatotoxicity in Batf3-KO mice was controlled by the adoptive transfer of WT hepatic CD103^+^ cDC1s into Batf3-KO mice but not by transferring WT hepatic CD11b^+^ cDC2s (Fig. 3e). These results suggest that hepatic CD103^+^ cDC1s play a crucial role in the control of APAP-ALI by inducing protective CD8^+^ T cells.

### Hepatic CD103^+^ cDC1/CD8^+^ T cell axis suppresses the accumulation of pathogenic Ly6C^hi^ monocytes in APAP-damaged liver

**Fig. 4:**
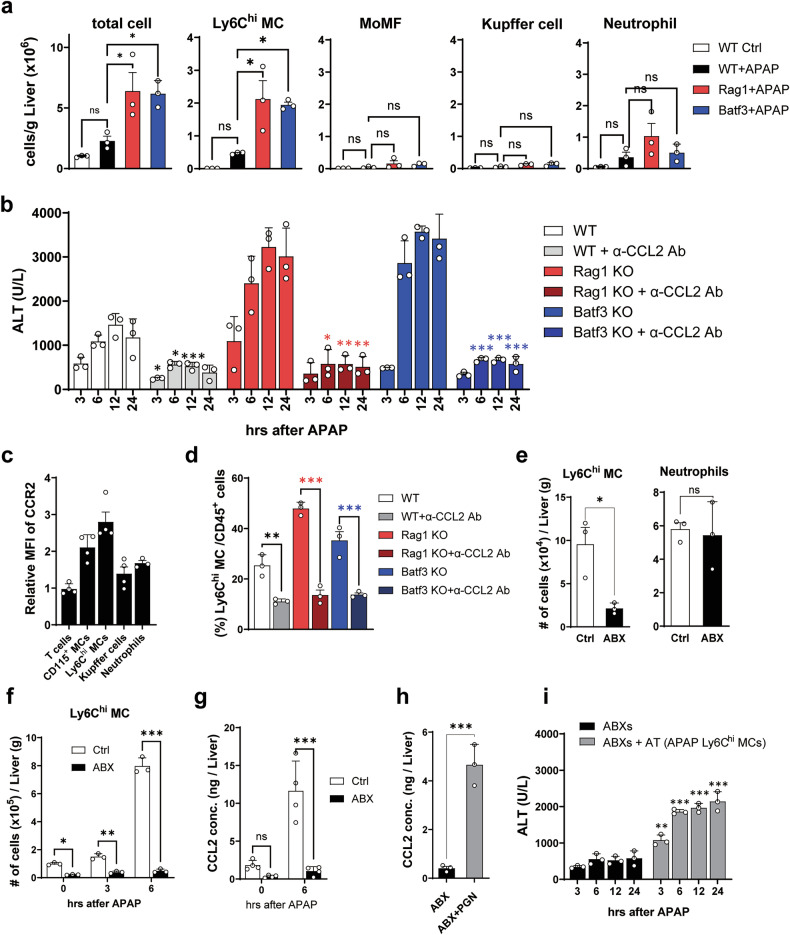
Hepatic CD103^+^ cDC1-derived CD8^+^ T cells inhibit Ly6C^hi^ monocyte accumulation. **a** The absolute number of liver immune cells in WT and KO mice 24 h after APAP. *n* = 3 per group. **b** The ALT level of WT and KO mice administrated with anti-CCL2 antibody before APAP. *n* = 3 per group per time point. **c** The relative CCR2 expression levels of liver immune cells analyzed by flow cytometry. *n* = 4 per group. **d** The frequency of Ly6C^hi^ monocytes in the liver of WT and KO APAP mice pretreated with anti-CCL2 neutralizing antibodies. *n* = 3 per group. **e** The absolute cell number of Ly6C^hi^ monocytes and neutrophils in normal and ABXs-treated mice without APAP. *n* = 3 per group. **f** The absolute cell number of Ly6C^hi^ monocytes in normal and ABXs-treated mice after APAP administration. *n* = 3 per group per time point. **g** The concentration of CCL2 in the liver of ABXs-pretreated APAP mice. *n* = 4 per group per time point. **h** The concentration of CCL2 in the liver of ABXs-pretreated WT mice 6 h after intraperitoneal injection of PGN. *n* = 3 per group. **i** The ALT level of ABXs-mice adoptively transferred with hepatic Ly6C^hi^ monocytes before APAP. *n* = 3 per group per time point. Ordinary one-way ANOVA with Dunnett in **a** and Tukey in **d** for the posttest, two-way ANOVA with Dunnett in **b** and Bonferroni in **f**, **g** and **i** for the posttest and unpaired two-tailed *t*-test in **e** and **h** were used to measure significance. The error bars indicate mean ± s.e.m. The black, blue and red asterisks represent the differences by time point compared with WT, Batf3-KO and Rag1-KO mice, respectively. ns, not significant. *P < 0.05, **P < 0.01, ***P *<* 0.001.

As shown in Fig. 1d, the transfer of hepatic immune cells from APAP mice to ABXs-treated APAP mice resulted in liver injury, indicating that the transferred hepatic immune cells harbor hepatotoxic immune cells. To explore APAP-induced hepatotoxic immune cells that would be regulated by hepatic CD103^+^ cDC1/CD8^+^ T cell axis, we examined various liver immune cell subsets, except cDCs and T cells, in WT, Rag1-KO and Batf3-KO APAP mice using a well-established immune cell gating strategy (Supplementary Fig. 2). The total number of immune cells, along with the number of Ly6C^hi^ monocytes in the liver, markedly increased in APAP mice, particularly in both KO APAP mice, but the numbers of other immune cells, such as MoMF, KC and neutrophils, did not show significant changes (Fig. 4a). It is well established that liver-infiltrating CCR2^+^ monocytes aggravate the early phase of APAP-ALI^9–11^. Here, we examined whether the increased number of Ly6C^hi^ monocytes in the liver of KO mice is directly associated with the worsening of APAP-ALI in KO mice or not. When mice were pretreated with the neutralizing antibody against CCL2, monocyte chemoattractant protein 1, ALT levels were significantly reduced in Batf3-KO and Rag1-KO APAP mice as well as in WT APAP mice (Fig. 4b). As anticipated, CCR2 expression was the most prominent in monocytes among the liver immune cells in APAP mice (Fig. 4c). Moreover, the frequency of Ly6C^hi^ monocytes in the liver was significantly reduced by blocking CCL2 in both WT and KO APAP mice (Fig. 4d). These findings suggest that the increased severity of APAP-ALI in KO mice can be attributed to the heightened number of Ly6C^hi^ monocytes infiltrated in the liver in KO APAP mice compared with WT APAP mice.

As aforementioned, APAP-ALI was alleviated by pretreatment of ABXs in both Rag1-KO mice and Batf3-KO mice as well as WT mice (Figs. 1, 2a and 3a). Even in a steady state, the number of Ly6C^hi^ monocytes significantly decreased in the liver of ABXs-treated mice compared with untreated control mice, whereas there were little differences in the number of neutrophils (Fig. 4e). After APAP administration, there was a rapid increase in the population of hepatic Ly6C^hi^ monocytes in WT mice, whereas ABXs-treated mice did not exhibit the increase (Fig. 4f and Supplementary Fig. 3a). Moreover, there were no significant differences in the number of liver neutrophils between WT and ABXs-treated mice after APAP administration (Supplementary Fig. 3b). It has been reported that hepatic MoMF or KC secrete CCL2 when exposed to DAMPs released from dying hepatocytes or other liver cells owing to the toxic effects of APAP overdose in the context of APAP-ALI^6^ or to the infection of hepatic pathogens^45^. In our experiments, we observed that the major population of hepatic immune cells expressing CCL2 was MoMF in APAP mice (Supplementary Fig. 3c). By contrast, the concentration of CCL2 significantly decreased in the liver of ABXs-treated mice and did not increase even after APAP administration (Fig. 4g). In addition, when ABXs-treated mice were injected with PGN, a pathogen-associated molecular pattern (PAMP) of G^+^ bacteria, a significant increase in CCL2 levels was observed in the liver of ABXs mice (Fig. 4h). Accordingly, there was a remarkable increase in the number of infiltrated Ly6C^hi^ monocytes (Supplementary Fig. 3d). However, the PGN administration alone did not induce liver damage (Supplementary Fig. 3e). These data suggest that G^+^ gut microbiota-derived PAMP triggers hepatic MoMF to express CCL2 in APAP mice, initiating Ly6C^hi^ MC infiltration, leading to the exacerbation of APAP-ALI.

Conversely, there was no clear difference in the expression of inflammatory cytokines in Ly6C^hi^ monocytes between ABXs-treated and untreated APAP mice (Supplementary Fig. 4). These findings indicate that G^+^ gut-microbiota-derived PAMP promotes the infiltration of blood Ly6C^hi^ monocytes into the liver by stimulating the hepatic MoMF to secret CCL2 rather than directly stimulating the inflammatory activity of Ly6C^hi^ monocytes. However, when Ly6C^hi^ monocytes from the liver of APAP mice were adoptively transferred into ABXs-treated mice, ALT levels increased upon APAP administration (Fig. 4i). Interestingly, the population of Ly6C^hi^ monocytes were found to be accumulated in the livers of recipient mice, even though CCL2 levels were lowered in the recipient mice due to ABXs pretreatments (Supplementary Fig. 5a). This result suggests that once localized in the liver after infiltration, the hepatic Ly6C^hi^ monocytes of APAP mice may exhibit CCL2-independent liver tropism. In an in vitro migration assay with liver extracts (Supplementary Fig. 5b), the migration of blood circulating Ly6C^hi^ monocytes was significantly blocked by CCL2 neutralizing antibody but that of hepatic Ly6C^hi^ monocytes of APAP mice was not (Supplementary Fig. 5c), indicating that, unlike blood circulating Ly6C^hi^ monocytes, hepatic Ly6C^hi^ monocytes may possess CCL2-independent homing properties. Nevertheless, the adoptive transfer of hepatic Ly6C^hi^ monocytes from APAP mice to APAP-untreated WT mice did not increase the ALT level in the recipient mice (Supplementary Fig. 6). It means that Ly6C^hi^ monocytes do not cause direct liver damage by themselves and are rather involved in exacerbating APAP-triggered liver injury.

### Hepatic CD8^+^ T cells induce apoptosis of liver infiltrated Ly6C^hi^ monocytes in APAP mice

**Fig. 5:**
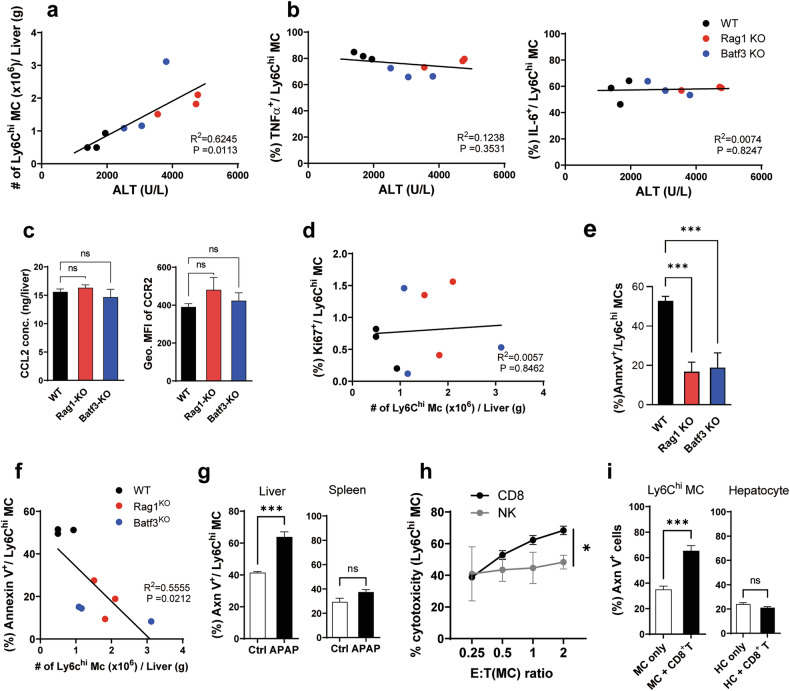
Hepatic CD8^+^ T cells induce apoptosis of Ly6C^hi^ monocytes. **a**, **b** The correlation between the ALT levels and the number of Ly6C^hi^ monocytes (**a**) and the frequency of TNFα^+^ or IL-6^+^ Ly6C^hi^ monocytes (**b**) in the liver of APAP mice. **c** The CCL2 concentrations in the liver and CCR2 expression on the hepatic Ly6C^hi^ monocytes of WT and KO APAP mice. *n* = 3 per group. **d** The correlation between the number of Ly6C^hi^ monocytes and the frequency of Ki67^+^ cells in the liver of WT and KO APAP mice. **e** The apoptotic cell death of Ly6C^hi^ monocytes in the liver of WT and KO APAP mice were assessed after annexin V and FVD staining. *n* = 3 per group. **f** The correlation between the number of Ly6C^hi^ monocytes and the frequency of annexin V^+^ cells in the liver of WT and KO APAP mice. **g** The frequency of annexin V^+^ Ly6C^hi^ monocytes in the liver and the spleen of APAP mice. *n* = 3 per group. **h** The cytotoxicity of hepatic CD8^+^ T cells and NK cells of APAP mice on hepatic Ly6C^hi^ monocytes as a target cells. *n* = 3 per group. **i** The apoptosis of Ly6C^hi^ monocytes (*n* = 6 per group) and hepatocytes (*n* = 3 per group) in the coculture with hepatic CD8^+^ T cells of APAP mice. Unpaired one-way ANOVA with Dunnett’s multiple comparisons test in **c** and **e**, unpaired two-tailed Student’s *t*-test in **g** and unpaired two-way ANOVA with Bonferroni for the posttest in **h** and **i** were used to measure significance. The error bars indicate mean ± s.e.m. ns, not significant. *P < 0.05, ***P < 0.001.

We next investigated the detailed control mechanisms of the hepatic CD103^+^ cDC1/CD8^+^ T cell axis on the pathogenic Ly6C^hi^-monocyte-mediated liver damages in APAP mice. First, we investigated the correlation between ALT levels in line with the number of Ly6C^hi^ monocytes together with their expression levels of liver damage-related proinflammatory cytokines such as TNFα and IL-6^9–11^. As expected, there was a strong positive correlation between ALT levels and the number of Ly6C^hi^ monocytes in the liver (Fig. 5a) but no correlation with their expression of TNFα or IL-6 (Fig. 5b). It means that the increased number of Ly6C^hi^ monocytes accumulated in the liver of Batf3-KO mice and Rag1-KO mice would be sufficient to exacerbate the liver injury more severely than WT mice, regardless of their inflammatory cytokine activity. We assumed three possible mechanisms for the increased number of Ly6C^hi^ monocytes in the liver of KO APAP mice compared with WT APAP mice; (1) Ly6C^hi^ monocyte infiltration was not properly controlled in KO mice; (2) Ly6C^hi^ monocyte expansion was not properly controlled in KO mice; or (3) Ly6C^hi^ monocyte cell death was not properly controlled in KO mice. We examined the CCL2 level in the liver and CCR2 expression of the Ly6C^hi^ monocytes of APAP mice and found that there was no significant difference between WT and KO mice (Fig. 5c). In addition, there was little correlation between the number of Ly6C^hi^ monocytes in the liver and their Ki67 expression level in both WT and KO APAP mice (Fig. 5d). By contrast, Ly6C^hi^ monocyte apoptosis was significantly reduced in the livers of both KO APAP mice compared with in WT APAP mice (Fig. 5e and Supplementary Fig. 7a). In addition, the increasing number of Ly6C^hi^ monocytes was inversely correlated with the rate of monocyte cell apoptosis (Fig. 5f). These results suggest that the increased number of Ly6C^hi^ monocytes in the liver of both KO APAP mice would be derived from the enhanced cell survival of Ly6C^hi^ monocytes rather than the enhanced infiltration or proliferation of Ly6C^hi^ monocytes. Interestingly, Ly6C^hi^ monocyte apoptosis significantly increased only in the liver of WT APAP mice but did not in the spleen (Fig. 5g). When hepatic Ly6C^hi^ monocytes were cocultured with hepatic CD8^+^ T cells of APAP mice, the Ly6C^hi^ monocytes were killed by hepatic CD8^+^ T cells in a dose-dependent manner but were little affected by hepatic NK cells (Fig. 5h). These results suggest that hepatic CD8^+^ T cells of APAP mice induce the apoptosis of the Ly6C^hi^ monocytes infiltrated into the liver of APAP mice. However, the hepatic CD8^+^ T cells in APAP mice did not cause damage to the hepatocytes (Fig. 5i and Supplementary Fig. 7b).

### T_RM_ cells within the hepatic CD8^+^ T cells play a key role in inducing apoptosis of Ly6C^hi^ monocytes in APAP mice through direct cell-to-cell interaction and GrzmB secretion

**Fig. 6:**
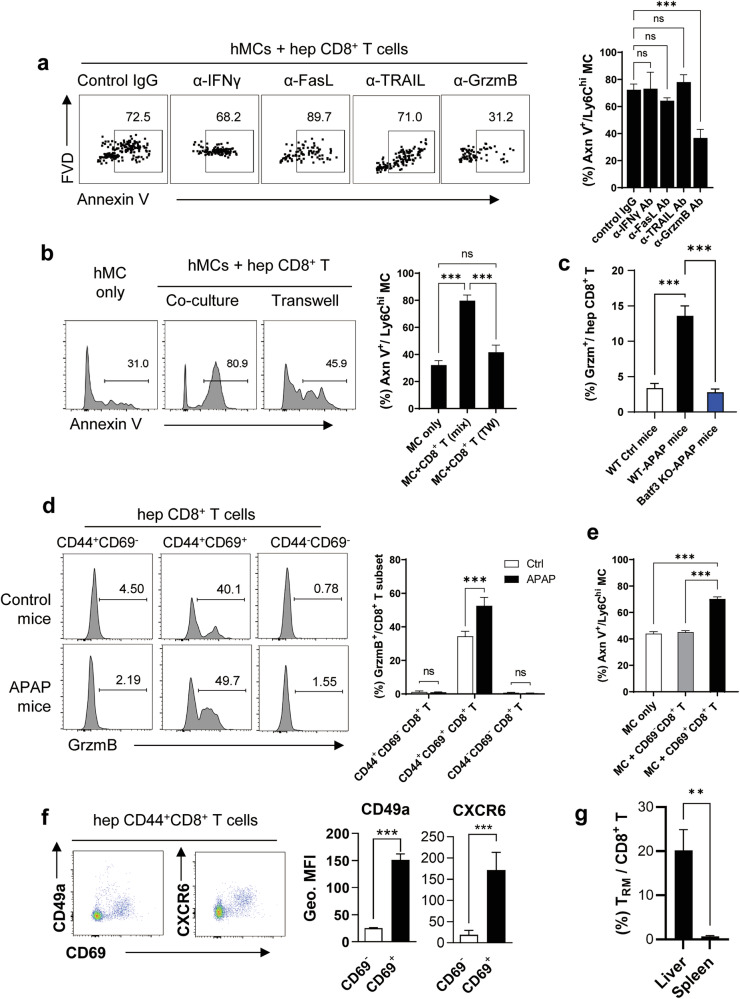
T_RM_ cells among the hepatic CD8^+^ T cells induce apoptosis of Ly6C^hi^ monocytes via cell-to-cell interaction and GrzmB secretion. **a**, **b** The apoptosis of Ly6C^hi^ monocytes in coculture with hepatic CD8^+^ T cells from APAP mice in the presence of each neutralizing antibody (*n* = 4 per group) (**a**) and in Transwell culture (*n* = 3 per group) (**b**). **c**, **d** The frequency of GrzmB^+^ cells in the hepatic CD8^+^ T cells of WT and Batf3-KO APAP mice (*n* = 3 per group) (**c**) and among the hepatic CD8^+^ T cell subsets in WT APAP mice (*n* = 3 per group) (**d**). **e** The apoptosis of hepatic Ly6C^hi^ monocytes when cocultured with hepatic CD69^−^CD8^+^ T cells or CD69^+^CD8^+^ T_RM_ cells. *n* = 3 per group. **f** The CD49a and CXCR6 expression was evaluated in CD69^−^CD8^+^ T cells and CD69^+^CD8^+^ T cells. *n* = 3 per group**. g** The frequency T_RM_ cells among the CD8^+^ T cells in the liver and spleen of WT mice. *n* = 3 per group. hep, hepatic. Ordinary one-way ANOVA with Dunnett in **a**, **c** and **e** and Tukey in **b** for the posttest and unpaired two-tailed Student’s *t*-test in **f** and **g** were used to measure significance. The error bars indicate mean ± s.e.m. ns, not significant. **P < 0.01, ***P < 0.001.

In further analysis, it was found that the apoptosis of hepatic Ly6C^hi^ monocytes by hepatic CD8^+^ T cells was significantly inhibited by the blocking antibody against GrzmB in the coculture condition, whereas it was little affected by other blocking antibodies against well-established monocyte apoptosis-inducing molecules, such as IFN-γ, Fas/Fas-ligand (Fas/FasL) and TRAIL^46,47^ (Fig. 6a). When cocultured with hepatic CD8^+^ T cells in a Transwell plate, hepatic Ly6C^hi^ monocyte apoptosis was significantly blocked (Fig. 6b). These results suggest that hepatic CD8^+^ T cells induce hepatic Ly6C^hi^ monocyte apoptosis via direct cell-to cell interaction. We have examined the cell adhesion molecules reported to be associated with the interaction between CD8^+^ T cells and monocytes, such as ICAM-LFA1 and CD44-CD44L^48,49^. However, when these adhesion molecules were inhibited by blocking antibodies, hepatic CD8^+^ T cell-mediated Ly6C^hi^ monocyte apoptosis was not affected (Supplementary Fig. 8). Interaction molecules between hepatic CD8^+^ T cells and Ly6C^hi^ monocytes remain to be further investigated. Conversely, the frequency of GrzmB^+^ hepatic CD8^+^ T cells was significantly increased in the liver of WT APAP mice but not in Batf3-KO APAP mice (Fig. 6c). Upon an in-depth analysis of hepatic CD8^+^ T cells in APAP mice, most of the GrzmB^+^CD8^+^ T cells were found in the population of CD44^+^CD69^+^ T_RM_ cells (Fig. 6d). When hepatic Ly6C^hi^ monocytes were cocultures with hepatic CD69^+^CD8^+^ and CD69^–^CD8^+^ T cells, CD69^+^CD8^+^ T_RM_ cells significantly induced apoptosis of Ly6C^hi^ monocytes, but CD69^–^CD8^+^ T cells did not (Fig. 6e). CD69^+^CD8^+^ T cells were further verified by the expression of additional T_RM_ markers, CXCR6 and CD49a (Fig. 6f). These findings suggest that the GrzmB^+^CD44^+^CD69^+^ T_RM_ cells are the hepatic CD8^+^ T cells responsible for inducing the apoptosis of hepatic Ly6C^hi^ monocytes in APAP mice. It is well known that T_RM_ cells predominantly reside in nonlymphoid organs or tissues rather than lymphoid organs^50^. As reported, a significantly higher frequency of T_RM_ cells was observed in the liver compared with the spleen of WT mice (Fig. 6g). This discrepancy in T_RM_ cell distribution is probably the reason why the adoptive transfer of hepatic CD8^+^ T cells, but not splenic CD8^+^ T cells, effectively controlled APAP-ALI in recipient Rag1-KO mice as shown in Fig. 2f.

### Bystander CD8^+^ T_RM_ cells activated by IL-15 secreted from hepatic cDC1s in APAP mice

**Fig. 7:**
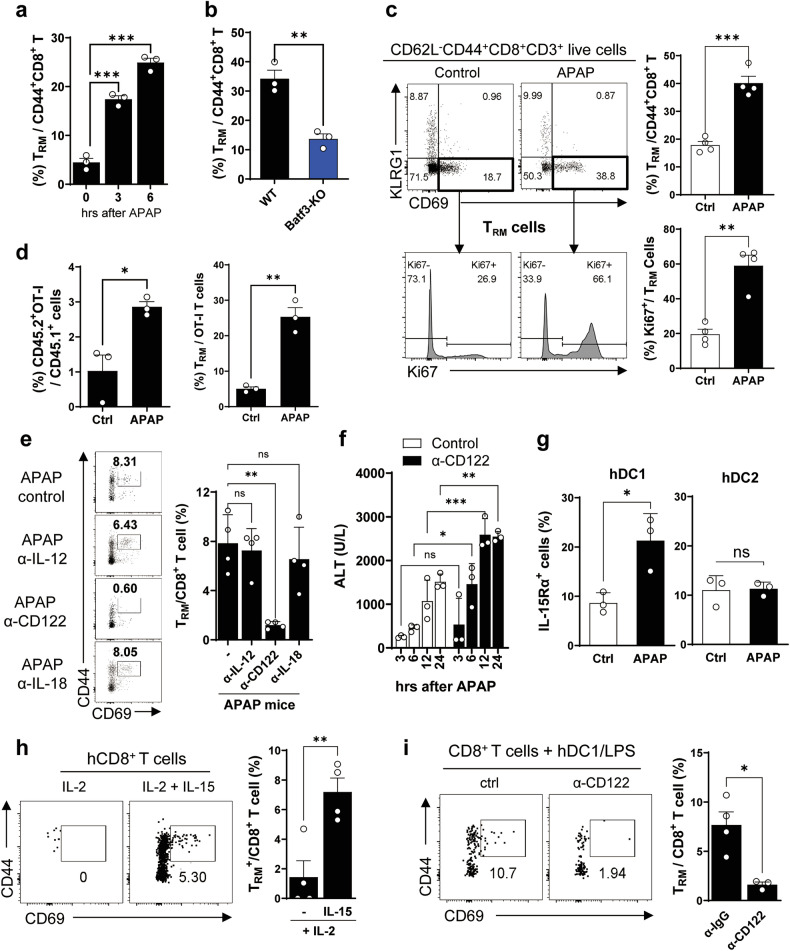
CD103^+^ cDC1s secret IL-15, which induces activation and proliferation of bystander CD8^+^ T_RM_ cells. **a**, **b** The frequency of hepatic CD69^+^ T_RM_ cells in WT APAP mice (*n* = 3 per group per time point) (**a**) and in WT and Batf3-KO mice 24 h after APAP administration (*n* = 3 per group) (**b**). **c** The frequency of Ki67^+^ cells in hepatic CD69^+^KLRG1^−^CD44^+^CD8^+^ T_RM_ cells in APAP mice. *n* = 4 per group. **d** The population of adoptively transferred OT-I T cells and of OT-I T_RM_ cells in the liver of recipient mice after APAP administration. *n* = 3 per group. **e** The frequency of CD69^+^ T_RM_ cells was assessed when APAP mice were pretreated with blocking antibodies against IL-12, IL-15 and IL-18. *n* = 4 per group. **f** The ALT level was assessed in APAP mice pretreated with IL-15 blocking antibodies (α-CD122). *n* = 3 per group per time point. **g** The frequency of IL-15Rα^+^ cells in the hepatic DC1s and DC2s of APAP mice. *n* = 3 per group. **h** The frequency of T_RM_ cells in hepatic CD8^+^ T cells when CD8^+^ T cells were cultured in the presence of IL-15. *n* = 4 per group. **i** The frequency of T_RM_ cells among CD8^+^ T cells when hepatic CD8^+^ T cells were cocultured with LPS-stimulated hepatic DC1s of APAP mice in the presence of IL-15 blocking antibodies. *n* = 4 per group. Ordinary one-way ANOVA with Dunnett for posttest in **a** and **e**, unpaired two-tailed Student’s *t*-test in **b**–**d** and **g**–**i** and two-way ANOVA with Bonferroni for posttest in **f** were used to measure significance. The error bars indicate mean ± s.e.m. ns, not significant. *P < 0.05, **P < 0.01, ***P < 0.001.

We further investigated CD44^+^CD69^+^CD8^+^ T_RM_ cells in APAP mice. Following APAP administration, the frequency of T_RM_ cells were significantly increased among the hepatic CD8^+^ T cells in WT mice (Fig. 7a) but not in Batf3-KO mice (Fig. 7b). To determine the cause of the increased T_RM_ population in the liver of WT APAP mice, we investigated cell infiltration, proliferation and differentiation. When mice were pretreated with FTY720 in drinking water and then administered with APAP, there was no difference in ALT level (Supplementary Fig. 9a), and the frequency of T_RM_ cells remained unchanged in the liver and liver draining lymph node (Supplementary Fig. 9b). These results suggest that the increased population of T_RM_ cells in the liver of APAP mice is not due to migration or infiltration from other lymphoid organs. Interestingly, after APAP administration, Ki67^+^ cells were significantly increased in CD69^+^ T_RM_ cells (Fig. 7c) compared with CD69^−^ effector T cells (Supplementary Fig. 9c). This result indicates that liver-resident T_RM_ cells rapidly proliferate in APAP mice. When adoptively transferred into WT mice, CD69^−^CD8^+^ T cells did not differentiate into CD69^+^ T_RM_ cells, and CD69^+^ T_RM_ cells did not lose CD69 expression in recipient mice after APAP administration (Supplementary Fig. 10). These results suggest that the increased population of T_RM_ cells in the liver of APAP mice is probably due to the rapid proliferation of liver-resident T_RM_ cells rather than the differentiation from other T cells or infiltration of the pre-existing T_RM_ cells from other lymphoid organs.

It is well known that CD8^+^ T cells are rapidly activated by appropriate cytokine even in the absence of cognate antigens^51,52^. To determine whether hepatic T_RM_ cells proliferate in an antigen-specific manner after an APAP administration, CD45.2^+^ hepatic OT-I T cells were adoptively transferred to CD45.1^+^ WT mice. OT-I T_RM_ cells significantly increased in the liver of recipient APAP mice even in the absence of OVA antigen (Fig. 7d). These results suggest that hepatic CD8^+^ T_RM_ cells in APAP mice can undergo bystander activation in the absence of cognate antigen. It has been reported that bystander CD8^+^ T_RM_ cells in patients with acute hepatitis A recognize target cells via NK-group 2D activating receptor (NKG2D) and its ligand interaction without T cell receptor engagement^53,54^. As reported, NKG2D expression was highly induced in hepatic CD8^+^ T_RM_ cells of APAP mice (Supplementary Fig. 11a). However, the blocking of NKG2D or other NKG2 family receptors on hepatic CD8^+^ T_RM_ cells did not affect the T_RM_-mediated hepatic monocyte apoptosis (Supplementary Fig. 11b), suggesting that NKG2D or related receptors are not involved in the hepatic T_RM_-mediated Ly6C^hi^ monocyte apoptosis.

We proceeded to investigate the cytokines involved in the bystander expansion of hepatic CD8^+^ T_RM_ cells among IL-12, IL-15 and IL-18, which are well known for their role in CD8^+^ T_RM_ cell activation in the liver^54,55^. Blocking antibodies against these cytokines revealed that IL-15 blockade using an anti-CD122 (IL-15Rβ) antibody significantly reduced the T_RM_ population in APAP mice, whereas blocking IL-12 or IL-18 had a minimal effect (Fig. 7e). Notably, serum ALT levels significantly increased in mice pretreated with anti-CD122 antibody before APAP administration (Fig. 7f). To assess whether hepatic cDC1s express IL-15, crucial for hepatic CD8^+^ T cell dependence, we examined IL-15Rα^+^ cells in DC fractions on the basis of the previous report on the trans-presentation of IL-15 via IL-15Rα^56,57^. Our findings showed a significant increase in the frequency of IL-15Rα^+^ cells in the hepatic cDC1 fraction in APAP mice, although the hepatic CD11b^+^ cDC2 fraction remained unaffected (Fig. 7g and Supplementary Fig. 12a). Further investigation into the factors involved in hepatic cDC1 activation to express IL-15 and IL-15Rα in the liver environments of APAP-ALI mice revealed that although APAP alone or APAP-induced hepatocyte damage did not induce the expression of IL-15Rα in DC1s, treatment with LPS significantly upregulated IL-15Rα expression in DC1s (Supplementary Fig. 12b). These results suggest that gut-microbiota-derived PAMP, rather than APAP-damaged hepatocyte-derived DAMPs, may activate hepatic cDC1s owing to increased intestinal permeability in the gut–liver axis in APAP-ALI mice as previously reported^58,59^. Furthermore, the population of T_RM_ cells significantly increased in hepatic CD8^+^ T cells when cultured in the presence of rmIL-15 (Fig. 7h), and the frequency of T_RM_ was completely reduced by IL-15 blockade even when cocultured with LPS-stimulated hepatic cDC1s (Fig. 7i). In the APAP-ALI model, liver injury typically develops within 24 h, and WT mice generally recover within a few days. Similarly, although Rag1-KO mice also experience severe liver injury, they eventually recover to a comparable extent as WT mice. To investigate whether the protective immune axis in the APAP-ALI model is temporally regulated or sustained, we rechallenged recovered mice with a second dose of APAP 1 week after the initial administration. Upon the second APAP challenge, WT mice exhibited markedly attenuated liver injury, whereas Rag1-KO mice showed liver injury that was similar to or even more severe than the first response (Supplementary Fig. 13). These results suggest that in WT mice, the T_RM_ cells induced by the protective immune axis during the initial APAP exposure contribute to the mitigation of liver injury upon re-exposure. By contrast, Rag1-KO mice lack T_RM_ cells, thus failing to mount a protective response upon the second APAP challenge. Taken together, our findings suggest that in APAP mice, (1) G^−^ gut microbiota-derived PAMPs, such as LPS, activate hepatic CD103^+^ cDC1s to express IL-15/IL-15Rα, (2) which induces the activation and proliferation of hepatic T_RM_ cells, (3) leading to the cell apoptosis of liver-infiltrating pathogenic Ly6C^hi^ monocytes through cell-to-cell interaction and GrzmB secretion. This hepatic CD103^+^ cDC1/CD8^+^ T_RM_/pathogenic monocyte apoptosis axis appears to play a crucial role in controlling the severity of APAP-ALI, and the resulting protective immune response is sustained at least for the time being.

### Human hepatic cDC1s also increase the population of hepatic T_RM_ cells through the expression of IL-15, thereby inducing the apoptosis of hepatic monocytes

**Fig. 8:**
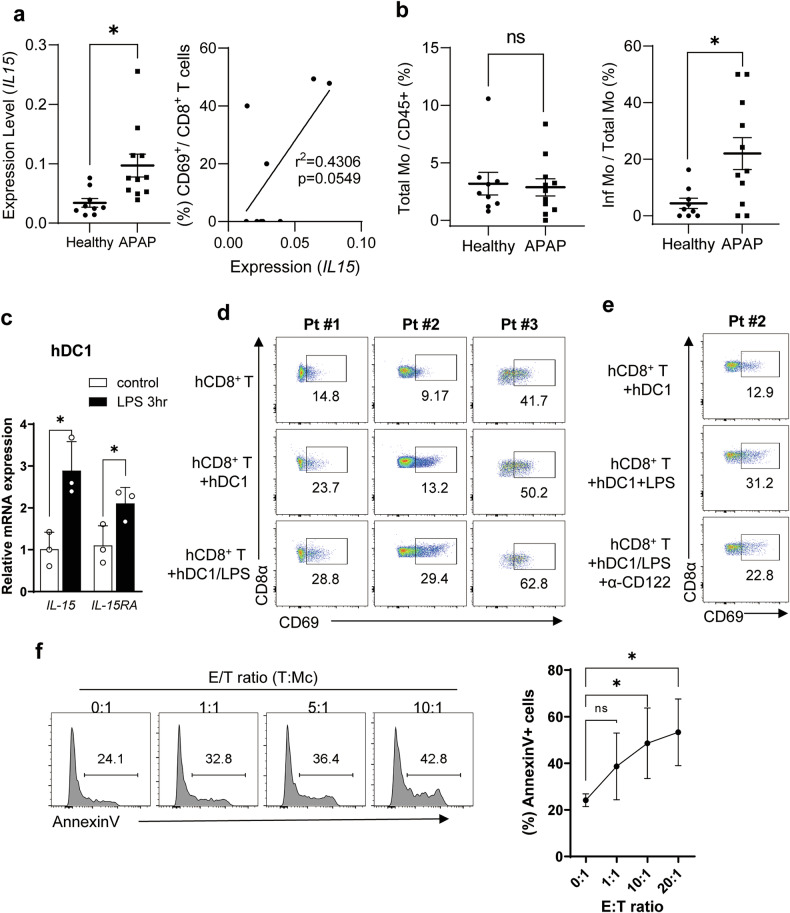
Human hepatic cDC1s activate hepatic T_RM_ cells through the expression of IL-15, inducing the apoptosis of hepatic inflammatory monocytes. **a** Based on the public single-cell RNA sequencing dataset from liver tissues of healthy donors and patients with APAP-ALI (GSE223581), the relative expression levels of IL-15 were assessed between APAP patients and healthy donors, and the correlation between IL-15 level and CD69^+^ T_RM_ population among the CD8^+^ T cells was evaluated. **b** Using the same dataset, the frequency of CD14⁺ monocytes among total liver immune cells (left) and the populaton of FCGR3A⁺ inflammatory monocytes among the CD14^+^ monocytes in the liver (right) were assessed between healthy donors and patients with APAP-ALI. For inflammatory monocytes, the ratio of FCGR3A >0.1 was assessed in the CD14 >0.1 population of monocytes. **c** The mRNA levels of *IL-15*/*IL-15RA* were assessed by quantitative real-time PCR in LPS-activated hepatic DC1s isolated from noncancerous healthy tissues of resected liver specimens obtained from patients with HCC and other liver diseases. *n* = 3 per group. **d**, **e** The frequency of human hepatic CD8^+^ T_RM_ cells when hepatic CD8^+^ T cells were cocultured with LPS-treated hepatic DC1s (**d**), in the presence of IL-15 blocking antibodies (α-CD122) (**e**). **f** The human hepatic inflammatory monocytes were cocultured with human hepatic T_RM_ cells at a different ratio, and the frequency of annexin V^+^ monocytes was assessed. *n* = 4 per group. Unpaired two-tailed Student’s *t*-test in **a** and **b**, multiple unpaired *t*-test in **c** and ordinary one-way ANOVA with Dunnett for the posttest **f** were used to measure significance. The error bars indicate mean ± s.e.m. ns, not significant. *P < 0.05.

We analyzed the relative expression levels of IL-15 in liver cells from APAP patients and healthy donors. We examined the correlation between *IL-15* messenger RNA (mRNA) level and CD69^+^ T_RM_ population among the CD8^+^ T cells using published single-cell RNA sequencing data from liver tissues of healthy donors and patients with APAP-ALI (GSE223581). Our findings reveal a significant increase in *IL-15* mRNA levels in patients with APAP-ALI compared with healthy donors and a positive correlation between *IL-15* mRNA level and T_RM_ population (Fig. 8a). In addition, although the overall frequency of CD14⁺ total monocytes in the liver did not significantly differ between healthy donors and patients with APAP-ALI, the proportion of FCGR3A⁺ inflammatory monocytes within the monocyte population was significantly increased in the livers of patients with APAP-ALI (Fig. 8b). Due to the limited availability of liver samples from patients with APAP-ALI, we investigated human liver immune cells isolated from noncancerous tissues of resected liver specimens obtained from patients with HCC and other liver diseases. The liver immune cells were isolated on the basis of the gating strategy illustrated in Supplementary Fig. 14a. We observed a significant increase in both IL-15 and IL-15RA mRNA levels in human hepatic cDC1s upon activation by LPS (Fig. 8c). Coculturing LPS-activated human hepatic cDC1s with hepatic CD8^+^ T cells resulted in an increase in the CD69^+^CD8^+^ T_RM_ population consistently in three different patient samples (Fig. 8d). This increase in hepatic cDC1-mediated T_RM_ population was also inhibited by blocking IL-15 with anti-CD122 antibody (Fig. 8e). Notably, these T_RM_ cells induced the apoptosis of CD16^+^CD14^+^ inflammatory hepatic monocytes, which were isolated from the liver immune cells on the gate (Supplementary Fig. 14b) as previously reported^60^, in a dose-dependent manner when cocultured (Fig. 8f). These results suggest the presence of a hepatic cDC1/T_RM_/monocyte apoptosis axis in the human liver, serving as a mechanism for liver protection against APAP-induced liver damage, consistent with the observations in experimental animal models.

## Discussion

APAP-ALI has been described in line with gut microbiota and immune cells^8–10,36,37^. However, the relationship between gut microbiota and immune responses in this context remains unclear. In this study, we found that the gut microbiota itself does not directly cause liver injury but triggers the infiltration of pathogenic monocytes, ultimately leading to APAP-ALI. Subsequently, we found that hepatic CD8^+^ T cells and cDC1s play a protective role rather than inducing liver injury in APAP-ALI model.

In the following mechanism studies, we found that (1) APAP overdose triggers liver injury, followed by G^+^ gut-microbiota-mediated upregulation of CCL2 expression within the liver, leading to the infiltration of Ly6C^hi^ monocytes from the bloodstream into the liver, thereby exacerbating liver injury. We also found that, (2) by contrast, APAP overdose also activates liver-resident CD103^+^ cDC1s via gut-microbial PAMP to secret IL-15, which in turn promotes the bystander activation of hepatic CD8^+^ T_RM_ cells that induced the apoptosis of Ly6C^hi^ monocytes infiltrated into the liver of APAP mice via cell-to-cell interactions and GrzmB secretion, thereby regulating APAP-ALI in APAP mice.

Although it is well established that Ly6C^hi^ monocytes play a key role in APAP-mediated liver injury^11^, there have been no reports on the intrahepatic immune system that regulates Ly6C^hi^ monocytes in APAP-ALI mouse model. In this study, we elucidated the existence of the hepatic CD103^+^ cDC1/ CD8^+^ T_RM_/ Ly6C^hi^ monocyte apoptosis axis as a protective immune system in the liver. This explains why the impairment of this protective immune system exacerbates APAP-ALI in Rag1-KO Batf3-KO mice.

We demonstrated that the gut microbiota increases the expression of CCL2 in the liver, leading to the infiltration of pathogenic Ly6C^hi^ monocytes and subsequent severe liver injury in APAP mice. However, the gut microbiota alone or the adoptive transfer of pathogenic Ly6C^hi^ monocytes themselves was insufficient to cause liver injury in normal mice. It became apparent that liver injury occurs only when APAP overdose triggers liver damage, and this injury is exacerbated by the cooperative action of G^+^ gut microbiota and/or derived PAMP and infiltrated pathogenic Ly6C^hi^ monocytes.

Batf3 gene deficiency has also been reported to affect T_reg_ differentiation^61^. However, the severity of APAP-ALI in Batf3-KO mice was markedly alleviated by the adoptive transfer of hepatic cDC1s from WT mice, suggesting that the severity of APAP-ALI in Batf3-KO mice is unlikely to be due to the enhancement of T_reg_. Previous studies have reported that Batf3-KO mice exhibit impaired memory transition of CD8⁺ T cells, resulting in a reduced number of CD8⁺ T_RM_ cells in the intestinal epithelium^62,63^. However, the marked improvement of APAP-ALI following the adoptive transfer of WT hepatic cDC1 into Batf3-KO mice (Fig. 3e) suggests that the exacerbated APAP-ALI in these mice is primarily attributable to cDC1 deficiency rather than impaired T_RM_ transition. If the aggravated liver injury were mainly due to T_RM_ loss caused by defective memory transition, the adoptive transfer of hepatic cDC1 would not have provided such protection.

APAP-ALI occurs within 24 h, which seemed too early to be associated with T cell-mediated adoptive immunity. Freeman et al.^51^ reported that CD8^+^ T cells have an ‘innate’ ability to be activated by cytokines in a T cell-receptor-independent pathway in the absence of cognate antigens. Indeed, OT-I T_RM_ cells were activated without the OVA protein when APAP-ALI was induced, suggesting that the CD8^+^ T_RM_ cells involved in APAP-ALI are activated in an antigen-nonspecific manner.

Recently, several reports have shown that IL-15 plays an important role in maintenance or activation of hepatic T_RM_ cells^28,64–66^. In the liver of hepatitis A virus (HAV)-infected patients, hepatic CD8^+^ T_RM_ cells are activated by IL-15 secreted from HAV-infected cells^53,55^. In APAP-ALI mice model, we found that hepatic cDC1s express IL-15, which is responsible for the expansion of intrahepatic bystander CD8^+^ T_RM_ cells. Interestingly, HAV-infected hepatocytes also express IL-15, promoting the generation of T_RM_ cells; however, these cells are known to contribute to liver damage. By contrast, hepatic T_RM_ cells activated by cDC1-derived IL-15 during APAP-ALI model selectively eliminate Ly6C^hi^ monocytes without damaging hepatocytes, thereby exerting a liver-protective effect. These findings suggest that although IL-15 is critical for bystander activation and differentiation of T_RM_ cells, additional environmental factors are likely to shape their functional properties. In other words, the tissue- or organ-specific microenvironment may play a decisive role in determining whether T_RM_ cells adopt pathogenic or protective functions, underscoring the need for further investigation into the context-dependent regulation of T_RM_ biology.

Bystander-activated CD8^+^ T_RM_ cells damage HAV-infected liver via NKG2D–ligand interaction^53,54^. Despite NKG2D upregulation in the T_RM_ cells of APAP mice, they did not damage hepatocytes, nor did blocking NKG2D or related receptors affect T_RM_-mediated monocyte apoptosis. In addition, FasL blocking did not suppress T_RM_-mediated apoptosis of Ly6C^hi^ monocytes, contrasting with prior reports of T cell-mediated hepatic damage via Fas-FasL^67^. Overall, our data indicate that APAP-induced hepatic-T_RM_-mediated monocyte apoptosis occurs through noncanonical cytotoxic pathways that are independent of NKG2D and FasL. Further research is needed to identify alternative death receptors or other cell-to-cell interaction molecules, such as integrin or C-type lectin, that mediate interactions between T_RM_ and Ly6C^hi^ monocytes and contribute to the associated cytotoxic mechanisms.

We observed that hepatic T_RM_ cells play a crucial role in controlling APAP-ALI in mice. However, during our investigation on the adoptive transfer of T_RM_ cells into Rag1-KO mice to assess the alleviation of ALI, we found that an excessive transfer of hepatic T_RM_ cells worsened liver injury instead of alleviating it (data not shown). This finding is consistent with a recent report on Capicua/ETS-KO mice, where an excessive expansion of T_RM_ cells led to ALI^68^. Our current findings demonstrated that hepatic T_RM_ cells in APAP mice serve a protective role in APAP-ALI by inducing the apoptosis of pathogenic monocytes. However, in certain circumstances, an excessive increase in the T_RM_ population may cause liver damage.

The liver-resident protective immune system, characterized by the hepatic cDC1/IL-15/T_RM_ proliferation/pathogenic monocyte apoptosis axis observed in the APAP-ALI mouse model, was likewise observed in the investigation of human liver immune cells.

In summary, it is widely recognized that APAP-ALI is amplified by pathogenic monocytes. Our study provides further insights by demonstrating that G^+^ gut microbiota or its derived PAMPs increase CCL2 expression in the liver, leading to an enhanced infiltration of pathogenic monocytes and subsequent development of APAP-ALI. Moreover, we have discovered a liver-specific protective immune system, the hepatic CD103^+^ cDC1/CD8^+^ T_RM_ axis. Upon activation in APAP mice, this immune system induces the apoptosis of liver-infiltrated pathogenic Ly6C^hi^ monocytes via cell-to-cell interaction and GrzmB secretion in APAP mice. This liver-specific immune system may play a crucial role in protecting the liver from ALI in APAP mice and humans as well. Consequently, targeting the activation of the hepatic CD103^+^ cDC1/CD8^+^ T_RM_ axis immune system could serve as another promising therapeutic strategy for APAP-mediated liver injury.

## Supplementary information

Supplementary Information
