# Supplementary material for: Discovery of intrahepatic CD103+ cDC1/CD8+ TRM protective immune axis against acetaminophen-induced acute liver injury
Source: Exp Mol Med. 2025 Nov 7;57(11):2458–74. doi: 10.1038/s12276-025-01565-3 (PMC12686067; doi:10.1038/s12276-025-01565-3)
Supplement: Supplementary file 1 — Supplementary Information [file 12276_2025_1565_MOESM1_ESM.pdf]

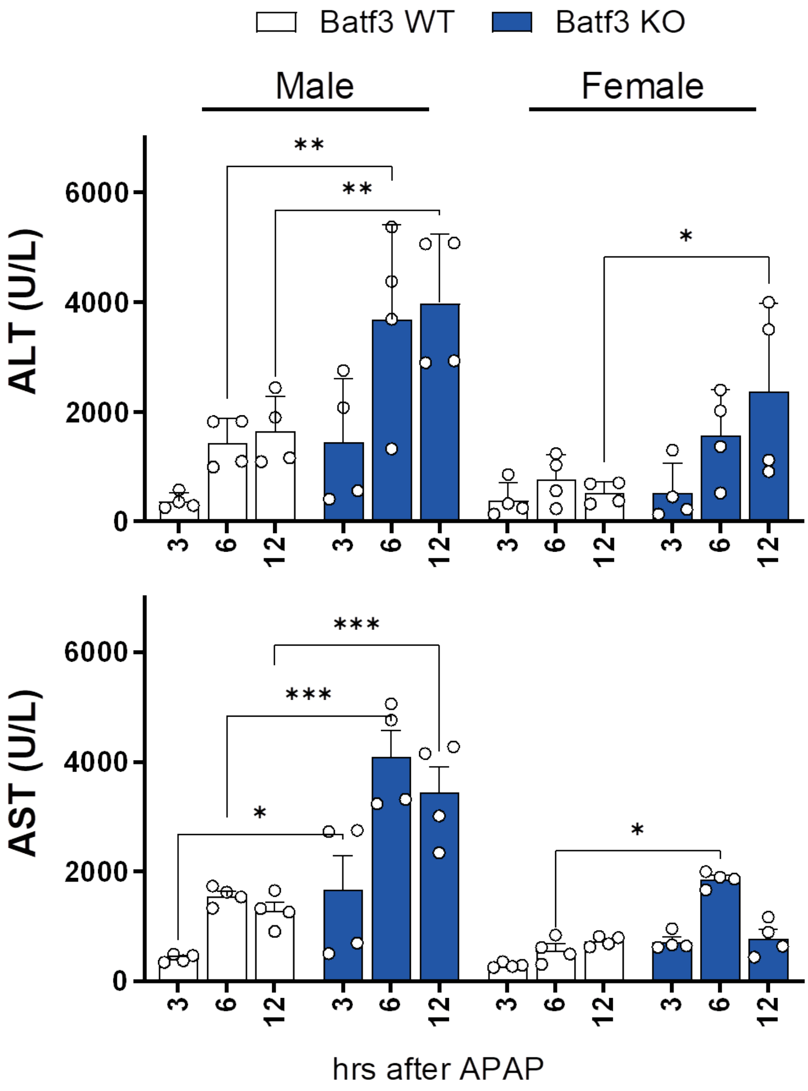

Supplementary Fig. 1 Batf3 KO mice show severe APAP-mediated acute liver injury regardless of gender. Serum ALT and AST levels of indicated time point were assessed after APAP treatment in mail and female WT and Batf3 KO mice. n = 4 per group per time point. Ordinary two-way ANOVA with tukey's multiple comparisons test, with individual variances computed for each comparison. *P < 0.05, **P < 0.01, ***P < 0.001; error bars indicate mean ± SD.

Gating strategy of hepatic innate immune cells

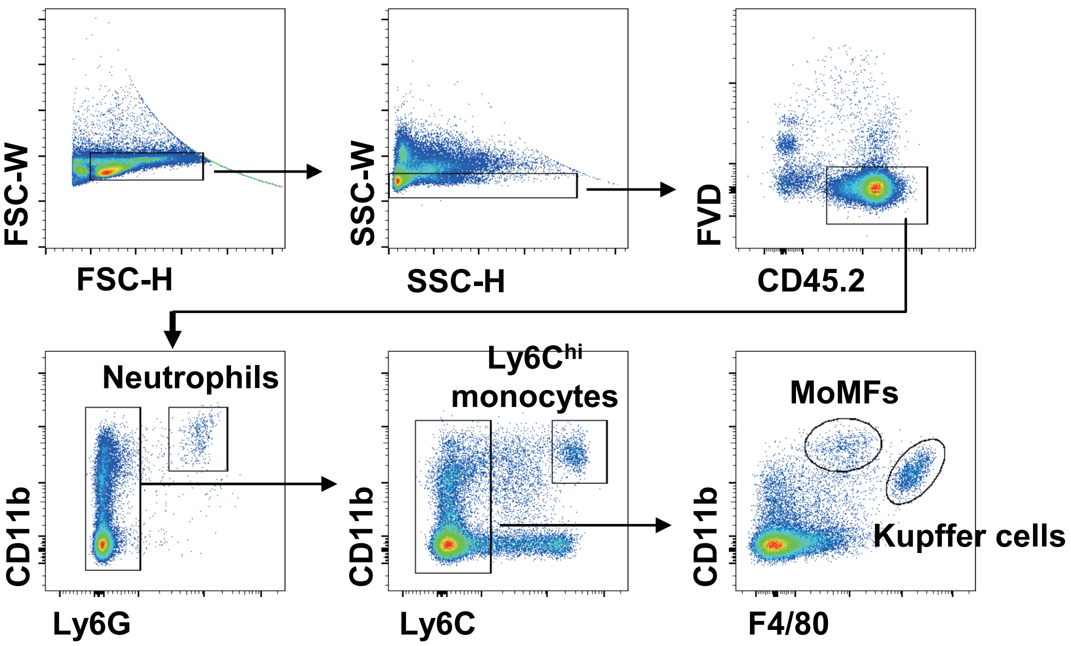

Supplementary Fig. 2 Gating strategy for hepatic innate immune cells in a steady state. Doublets dead cells excluded using FSC-W vs FSC-H, SSC-W vs SSC-H and FVD. Identification of four subpopulations in CD45.2^{+} immune cells: CD11b^{+}Ly6G^{+} neutrophils, Ly6G^{-}CD11b^{+}Ly6C^{+} Ly6C^{hi} monocytes, Ly6G^{-}Ly6C^{-} CD11b^{+}F4/80^{int} monocyte-derived macrophages (MoMFs) and Ly6G^{-}Ly6C^{-} CD11b^{int}F4/80^{+} Kupffer cells.

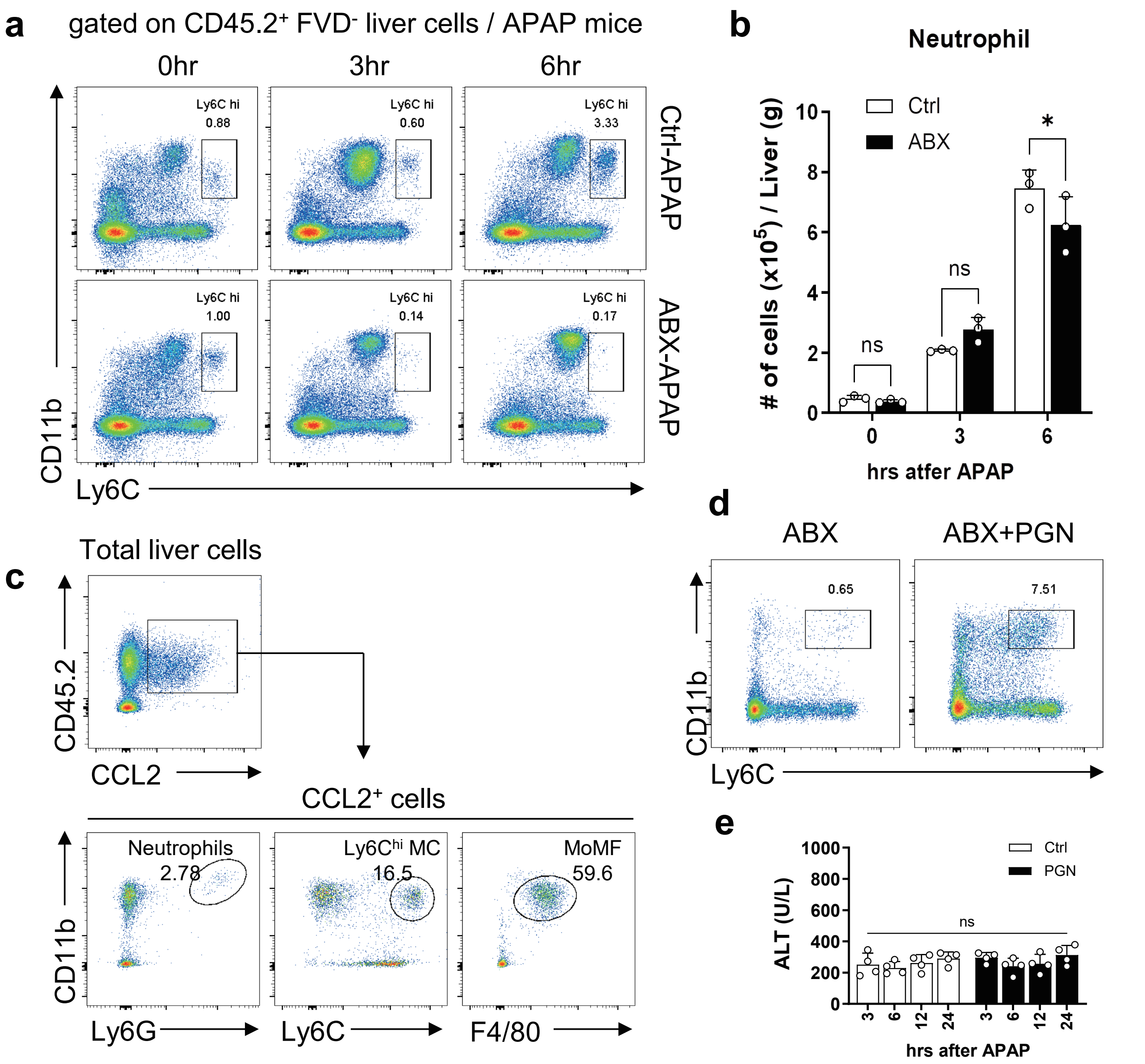

Supplementary Fig. 3 Gut microbiota-derived PAMP increases the infiltration of Ly6C^{hi} monocytes into the liver via MoMF-derived CCL2, even in ABX-treated mice. a-b, ABX-treated (for 2 weeks) or untreated mice were administered with APAP, and liver immune cells were assessed at 0, 3, 6hr after APAP treatment. **(a)** Hepatic Ly6C^{hi} monocytes were evaluated (representative FACS data for Fig. 4f). **(b)** The absolute cell number of neutrophils. n = 3 per group per time point. **c**, CCL2-expressing immune cells in the liver 6hr after APAP administration. **d**, The population of Ly6C^{hi} monocytes in the liver of ABX-pretreated mice 6hr after PGN inoculation or not. **e** Serum ALT levels were assessed in WT mice after PGN inoculation. Unpaired two-way ANOVA with bonferroni for post-test (b) was used to measure significance. ns, not significant; error bars indicate mean ± SD.

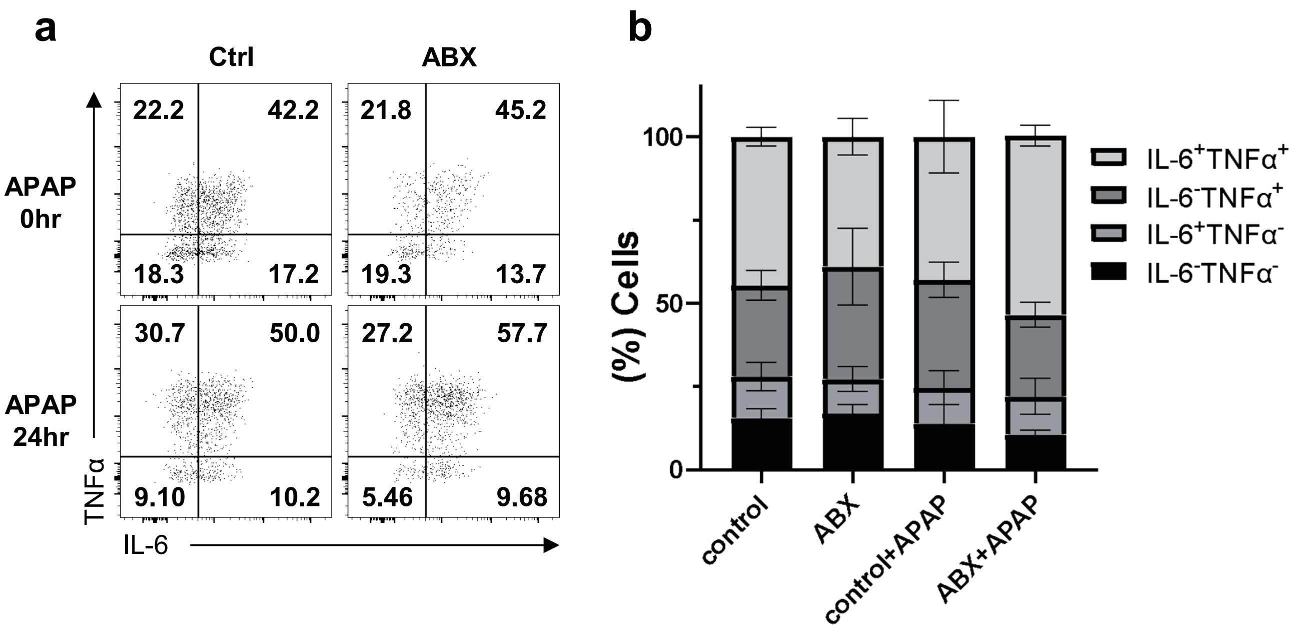

Supplementary Fig. 4 Gut microbiota does not affect Ly6C^{hi} monocyte activity. **a**, Flow cytometry analysis of IL-6^{+} and/or TNFα^{+} Ly6C^{hi} monocytes in the liver of ABX-treated or untreated mice at 0hr and 24hr after APAP treatment. **b**, Statistical analysis of Ly6C^{hi} monocytes expressing IL-6 and/or TNFα. n = 3 per group.

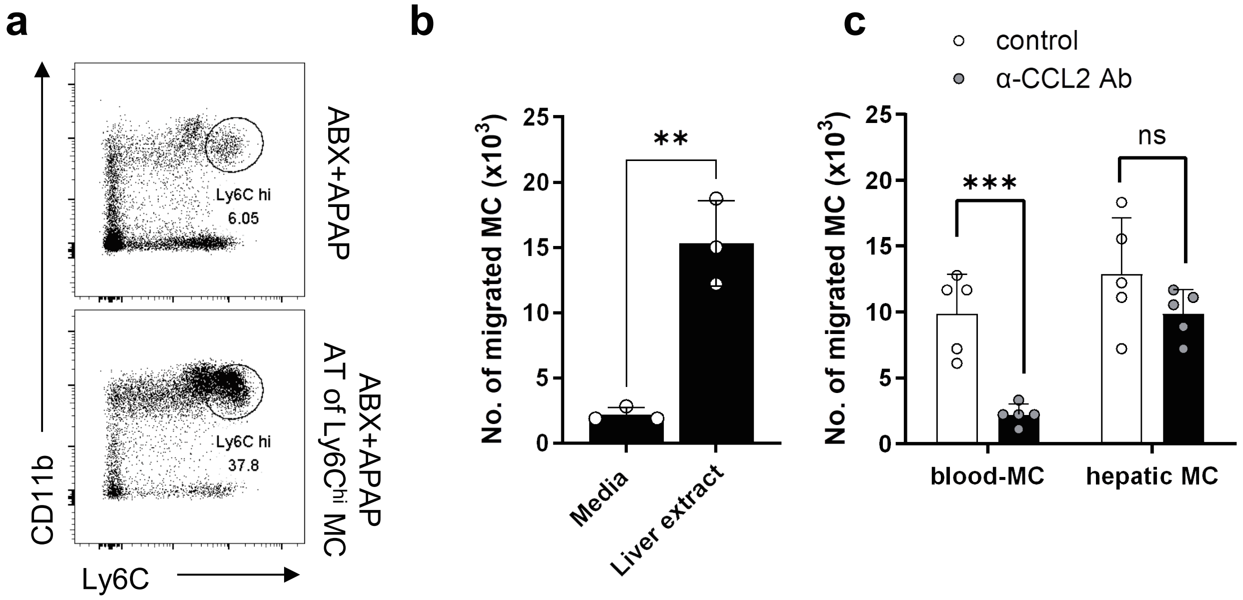

Supplementary Fig. 5 Once infiltrated and residing, hepatic Ly6C^{hi} monocytes have liver tropism in a CCL2-independent manner. a, Frequency of Ly6C^{hi} monocytes in the liver of ABX-treated APAP recipient mice after adoptive transfer (AT) of hepatic Ly6C^{hi} monocytes from APAP-ALI mice. **b-c**, Transwell migration assay: (**b**) Numbers of hepatic Ly6C^{hi} monocytes migrated into the liver extract. (**c**) Number of blood Ly6C^{hi} monocytes and hepatic Ly6C^{hi} monocytes migrated into the liver extract with or without CCL2 neutralizing Ab. Unpaired two-tailed student's t-test (b,c) was used to measure significance. **P < 0.01, ***P < 0.001, ns, not significant; error bars indicate mean ± SD.

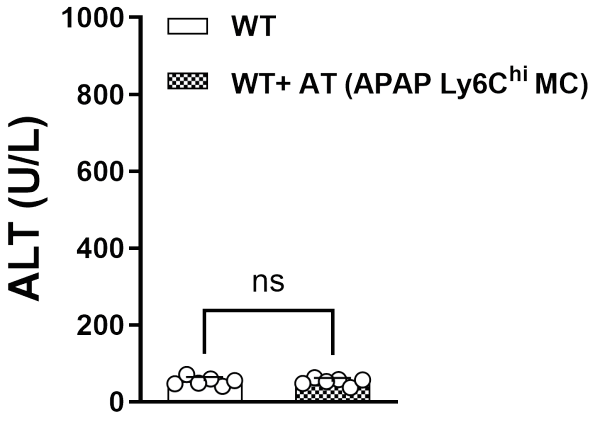

Supplementary Fig. 6 Ly6C^{hi} monocytes are not the sole factor contributing to liver injury. Hepatic Ly6C^{hi} monocytes isolated from APAP-ALI mice were adoptively transferred into APAP-untreated normal mice. Serum ALT level was evaluated in the recipient mice 24hr after adoptive transfer of Ly6C^{hi} monocytes. n = 6 per group. Unpaired two-tailed student's t-test was used to measure significance. ns, not significant; error bars indicate mean ± SD.

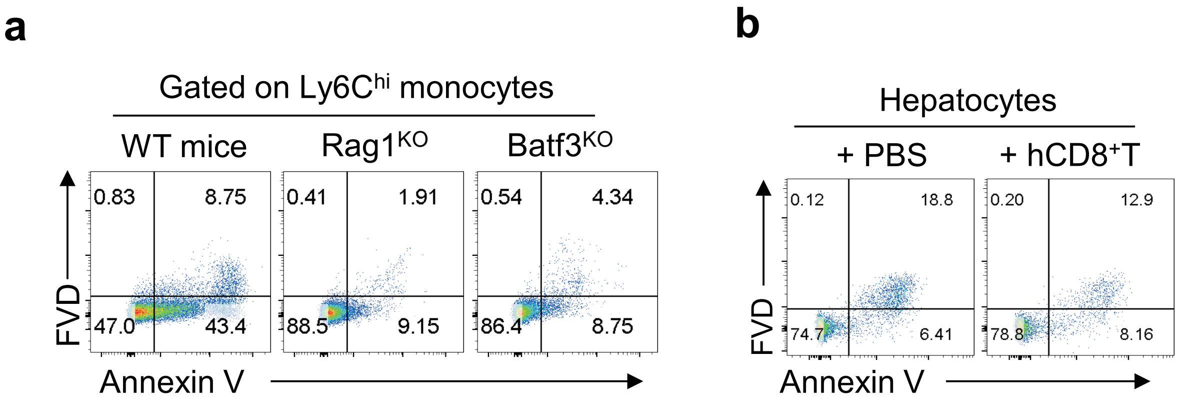

Supplementary Fig. 7 Apoptotic cell death of Ly6C^{hi} monocytes and hepatocyte. a, Apoptotic cell death of Ly6C^{hi} monocytes in the liver of WT and KO APAP mice was assessed after Annexin V and FVD staining. (Representative FACS data of Fig. 5e) **b.** Hepatocytes from WT mice were co-cultured with hepatic CD8^{+} T cells (hCD8^{+} T) of APAP mice. After 15hr, apoptotic hepatocytes were assessed after Annexin V and FVD staining. (Representative FACS data of Fig. 5i).

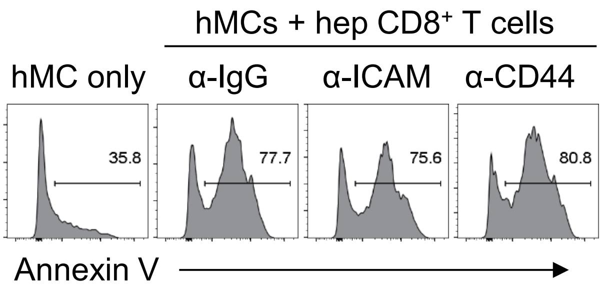

Supplementary Fig. 8, Hepatic Ly6C^{hi} monocytes (hMCs) were co-cultured with hepatic CD8^{+} T cells for 15 hrs in the presence of blocking antibodies against cell adhesion molecules, and the hMC apoptosis was examined after Annexin V and FVD staining.

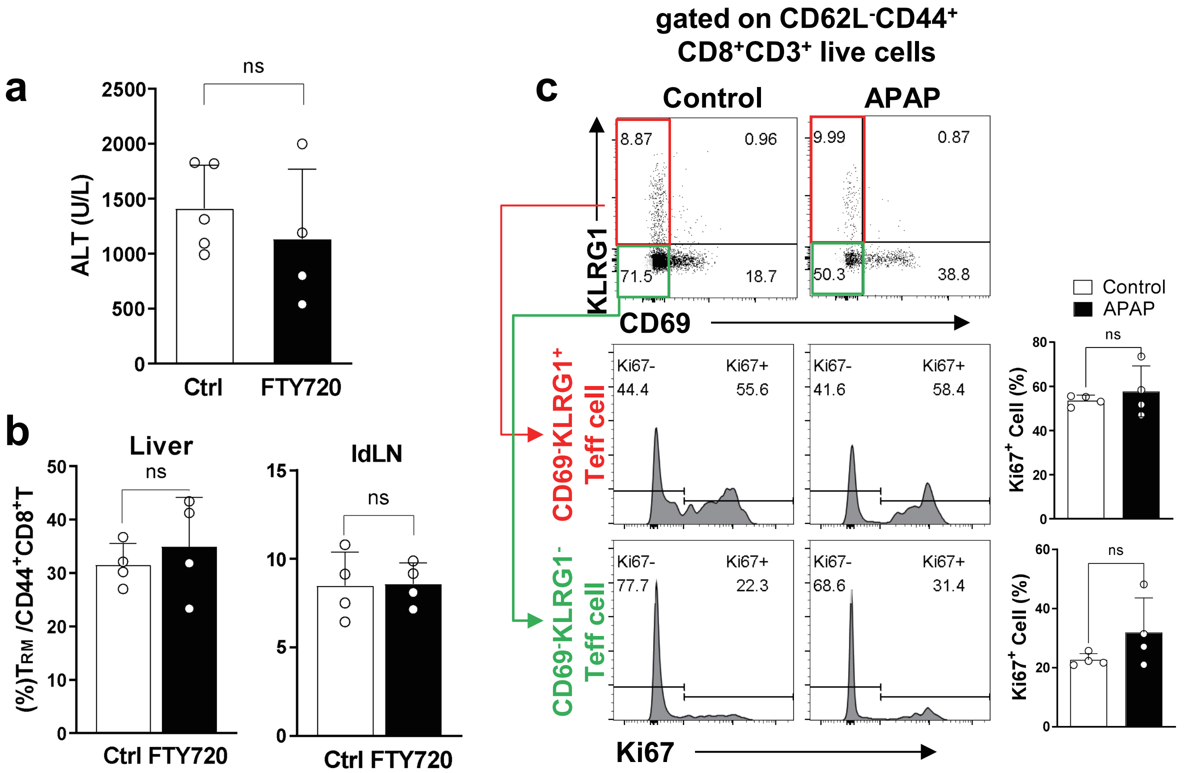

Supplementary Fig. 9 Increased population of hepatic CD8⁺ T_{RM} cells in the liver of APAP mice is not due to the enhanced migration or infiltration from other organ or tissue in APAP mice. a-b, Mice were pre-treated with FTY720 in the drinking water for 2 weeks and then administered with APAP. n = 4-5 per group. **a**, Serum ALT level 6hr after APAP administration. **b**, Frequency of T_{RM} cells in $CD44^+CD8^+$ T cells in the liver and liver draining lymph node (ldLN). n = 4 per group. **c**, Hepatic $CD69^+CD8^+$ T cells were evaluated for their proliferating capacity in main Fig. 6c. Here, $CD69^-CD8^+$ T cells were divided into $KLRG1^+CD44^+CD8^+$ T cell (red) and $KLRG1^-CD44^+CD8^+$ T cell (green), and their proliferating capacities were evaluated with Ki67 staining. n = 4 per group. Unpaired two-tailed t-test (a-c), ns, not significant; error bars indicate mean ± SD.

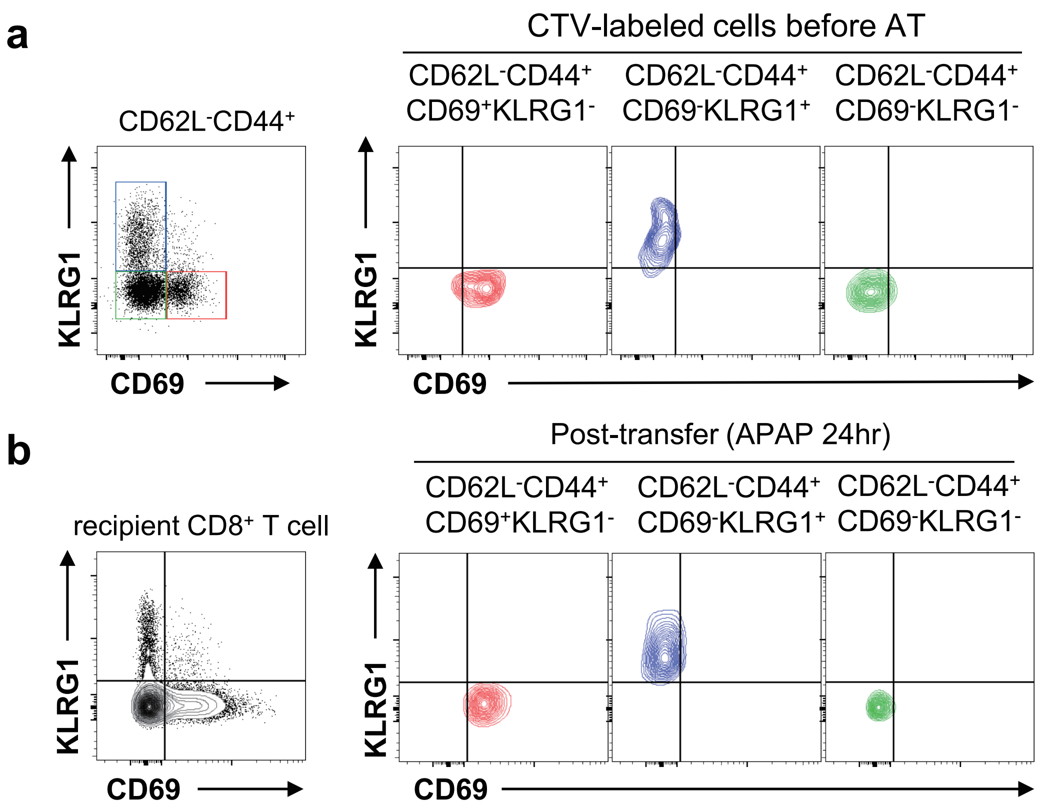

Supplementary Fig. 10 Hepatic $CD8^+CD69^+$ T_{RM} cells are not derived from $CD69^-$ T_{eff} cells after APAP administration. In APAP-ALI mice, hepatic $CD62L^-CD44^+CD8^+$ T cells were further divided into $CD69^+KLRG1^-$(red), $CD69^-KLRG1^+$(blue) and $CD69^-KLRG1^-$(green). Each fraction was labeled with CTV, and the cells were adoptively transferred into WT recipient mice through *i.v.*. Recipient mice were treated with APAP, and 24 hr later, the phenotype of the donor cells was analyzed. Before (**a**) and after (**b**) adoptive transfer, CD69 and KLRG1 expression of CTV-labeled donor cells was evaluated.

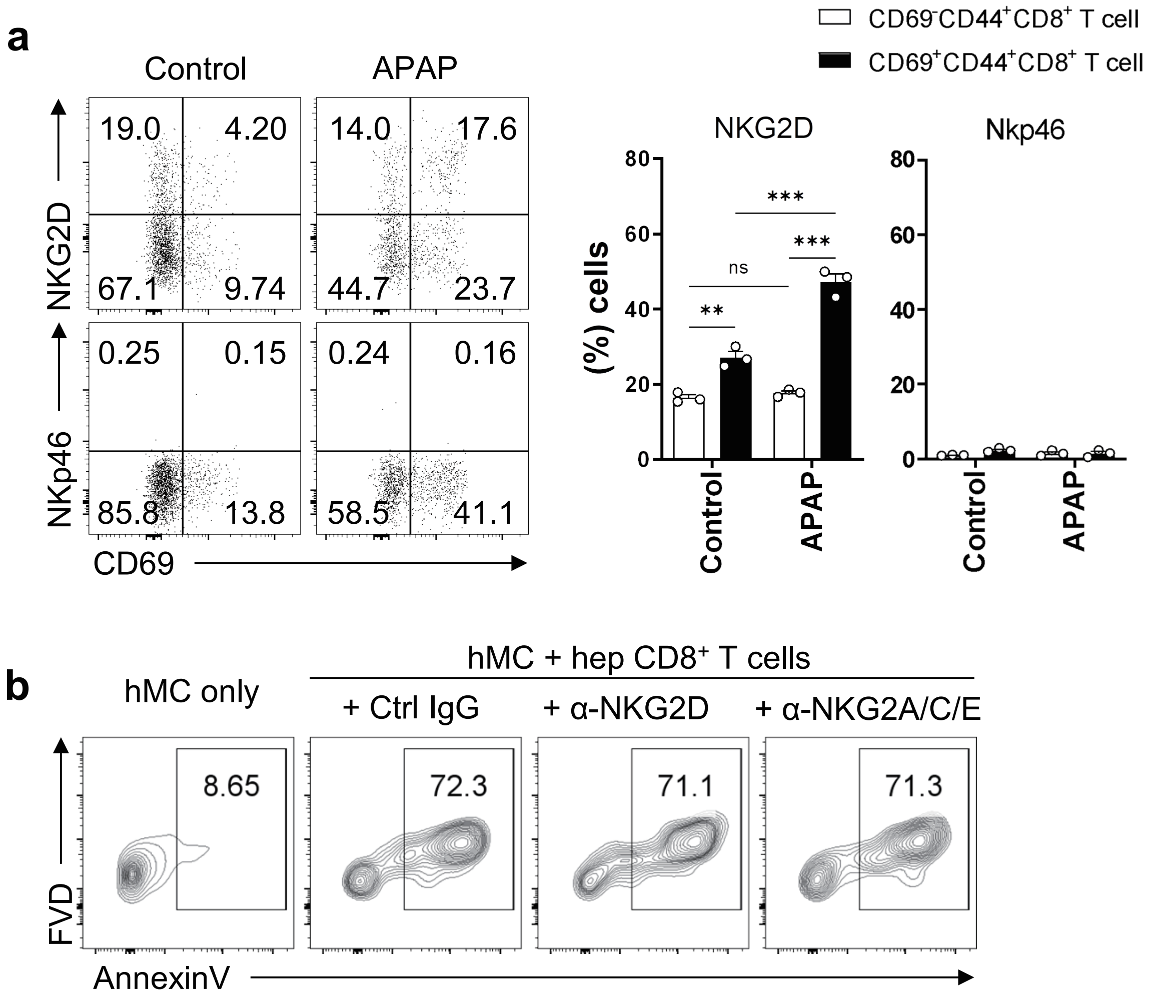

Supplementary Fig. 11 The apoptosis of hepatic Ly6C^{hi} monocytes in APAP-ALI mice is not associated with NKG2D expression in hepatic CD8^{+} T cells. a, The expression of NKG2D and NKp46 was examined in hepatic CD44^{+}CD8^{+} T cells in control mice and APAP-ALI mice. Frequency of NKG2D^{+} cells and NKp46^{+} cells were examined in hepatic CD69^{+} or CD69^{-} CD8+ T cells. n = 3 per group. **b**, Hepatic Ly6C^{hi} monocytes (hMCs) were co-culture with hepatic CD8^{+} T cells in the presence of control IgG, anti-NKG2D or anti-NKG2A/C/E blocking antibodies, and then apoptotic cell death of hMCs was evaluated by Annexin V and FVD staining. Unpaired two-way ANOVA with tukey for post-test (a) was used to measure significance. **P < 0.01, ***P < 0.001, ns, not significant; error bars indicate mean ± SD.

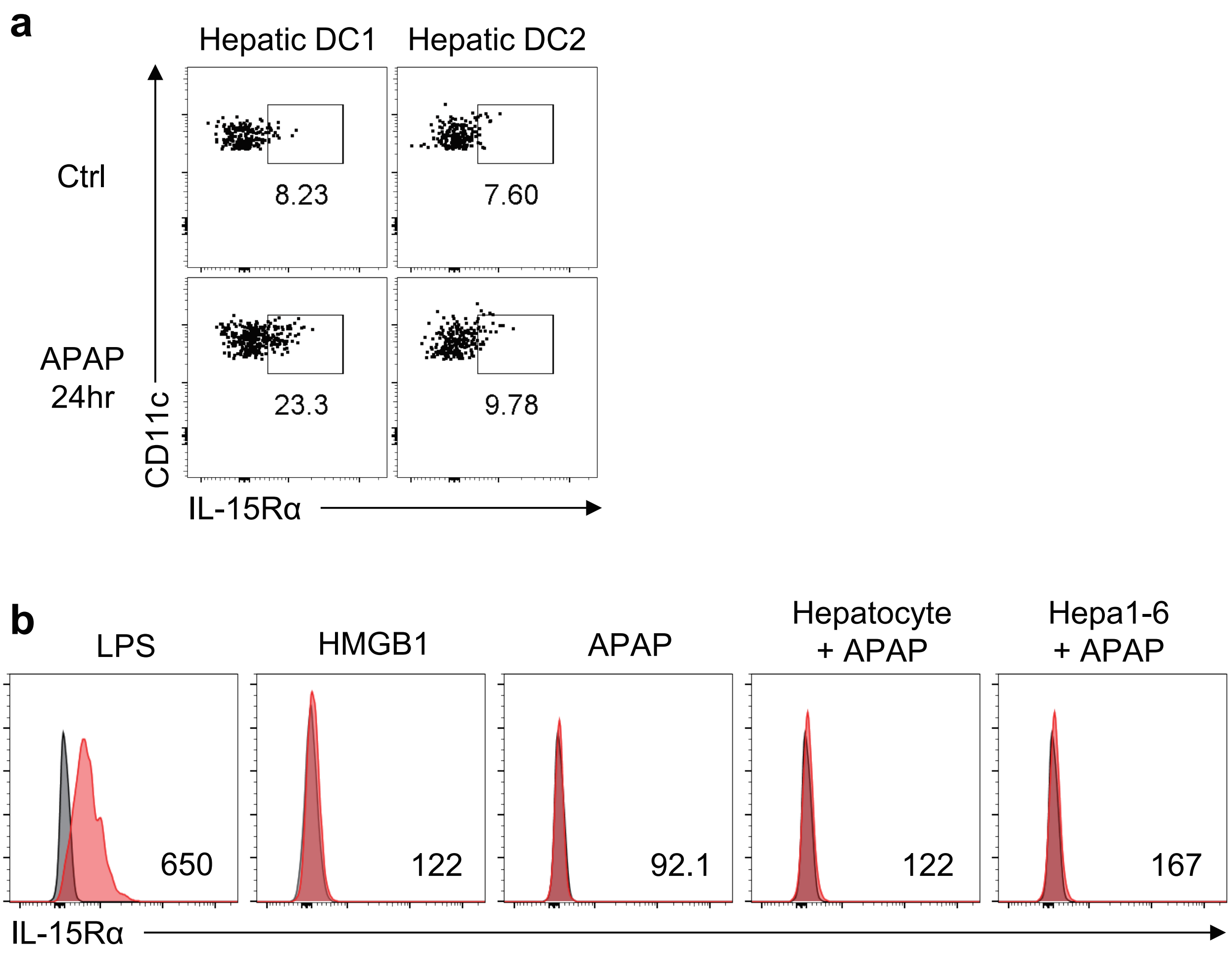

Supplementary Fig. 12 IL-15Rα expression in hepatic DCs in APAP mice. a, Hepatic cDC1 and cDC2 form WT and APAP mice were examined for the expression of IL-15Rα for alternative quantitation of IL-15 expressing cells (Representative FACS data for main Fig. 6g). **b**, PAMP or DAMP effects on IL-15 expression in hepatic cDC1s. Hepatic cDC1s were treated with LPS (1ug/ml), HMGB1 (500ng/ml), APAP alone (6μM) or the supernatants of APAP-treated mouse hepatocytes and APAP-treated Hepa1-6 cell line for 24 hrs. Surface expression of IL-15Rα was assessed by FACS.

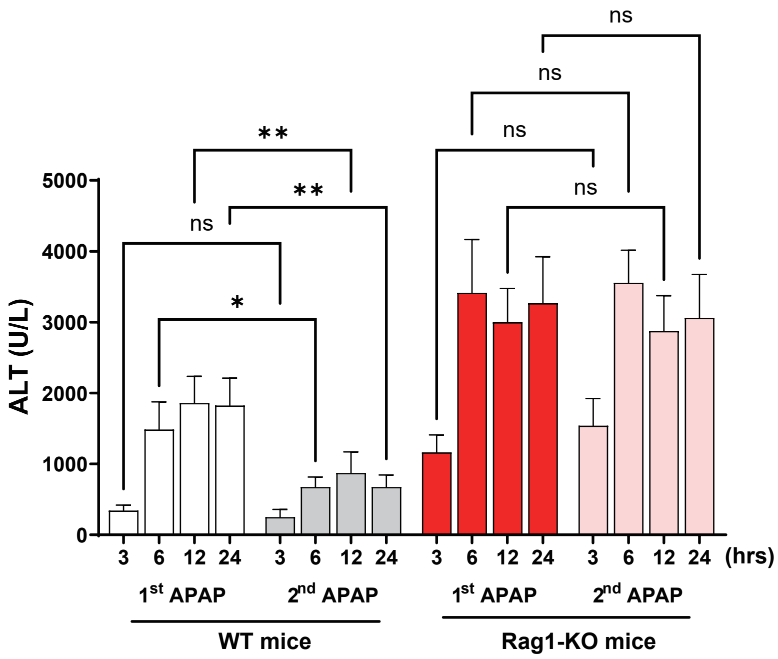

Supplementary Fig. 13. Serum ALT levels in WT and Rag1-KO mice administered with APAP in a week interval. WT (n= 4) and Rag1-KO (n =4) mice were administered 1st with APAP, and ALT levels were assessed per time point. A week later these mice were secondary administered with APAP, and ALT levels were assessed. Unpaired two-way ANOVA with Tukey for post-test was used to measure significance. *P < 0.05, **P < 0.01; error bars indicate mean ± SD.

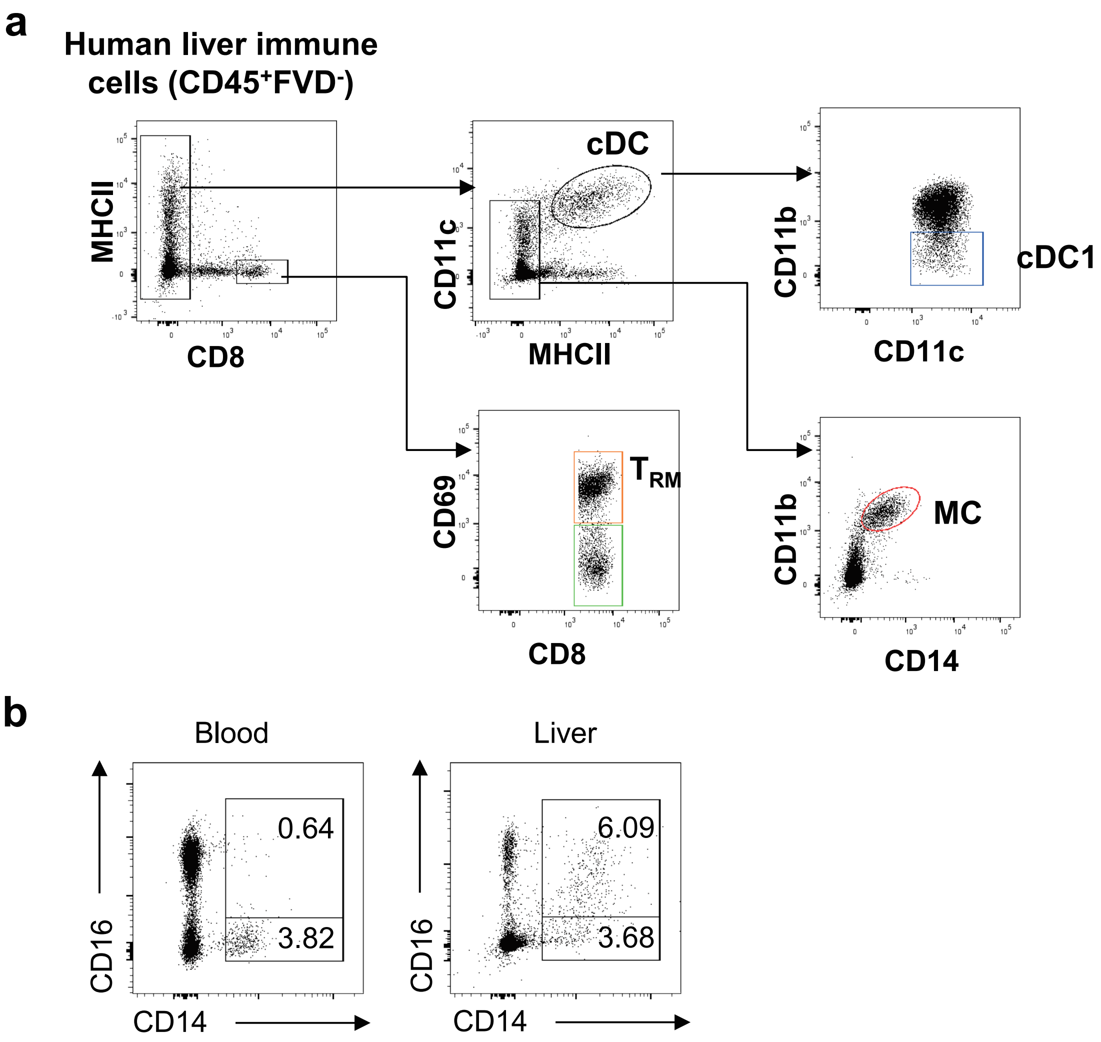

Supplementary Fig. 14 Gating strategy for human liver immune cells. a, Gating strategy for human hepatic cDC1s, monocytes (MC) and T_{RM} cells from the human hepatic $CD45^+$ immune cells isolated from resected liver samples of HCC patients**.** Doublets dead cells excluded using FSC-W vs FSC-A, SSC-W vs SSC-A and FVD. **b**, $CD16^+CD14^+$ inflammatory monocytes were assessed in peripheral blood and in the liver.
